# SIRT6 Is Essential for Adipocyte Differentiation by Regulating Mitotic Clonal Expansion

**DOI:** 10.1016/j.celrep.2017.03.006

**Published:** 2017-03-28

**Authors:** Qiang Chen, Wenhui Hao, Cuiying Xiao, Ruihong Wang, Xiaoling Xu, Huiyan Lu, Weiping Chen, Chu-Xia Deng

**Affiliations:** 1 Faculty of Health Sciences, University of Macau, Macau SAR, China; 2 Genetics of Development and Disease Branch; 3 Genomic Core Laboratory, National Institute of Diabetes and Digestive and Kidney Diseases, NIH, Bethesda, MD 20892, USA; 4 Lead Contact

## Abstract

Preadipocytes initiate differentiation into adipocytes through a cascade of events. Mitotic clonal expansion, as one of the earliest events, is essential for adipogenesis. However, the underlying mechanisms that regulate mitotic clonal expansion remain elusive. SIRT6 is a member of the evolutionarily conserved sirtuin family of nicotinamide adenine dinucleotide (NAD)+-dependent protein deacetylases. Here, we show that SIRT6 deficiency in preadipocytes blocks their adipogenesis. Analysis of gene expression during adipogenesis reveals that KIF5C, which belongs to the kinesin family, is negatively regulated by SIRT6. Furthermore, we show that KIF5C is a negative factor for adipogenesis through interacting with CK2α′, a catalytic subunit of CK2. This interaction blocks CK2α′ nuclear translocation and CK2 kinase activity and inhibits mitotic clonal expansion during adipogenesis. These findings reveal a crucial role of SIRT6 in adipogenesis and provide potential therapeutic targets for obesity.

## In Brief

Chen et al. demonstrate that SIRT6 is essential for adipogenesis by regulating mitotic clonal expansion. KIF5C is negatively regulated by SIRT6, and KIF5C is a negative regulator for adipogenesis by blocking CK2α′ nuclear translocation and CK2 kinase activity.

## Graphical Abstract

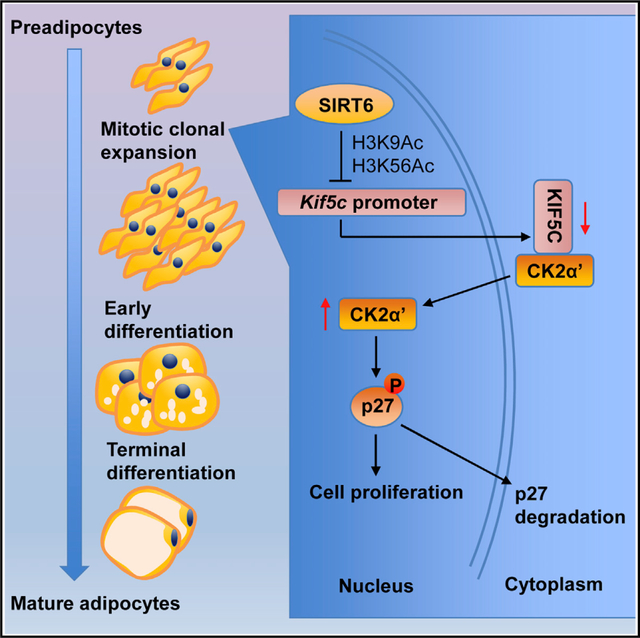

## INTRODUCTION

Adipocytes are derived from pluripotent mesenchymal stem cells (MSCs) that become restricted to the adipocyte lineage through a multistep process of commitment (Tang and Lane, 2012). Recruitment to this lineage gives rise to preadipocytes, which undergo several stages and then differentiate into adipocytes. One of the earliest events occurring in adipogenesis is that growth-arrested preadipocytes synchronously re-enter the cell cycle and undergo several rounds of cell divisions, known as mitotic clonal expansion (Tang et al., 2003b). It is indicated that mitotic clonal expansion is essential for adipogenesis, and C/EBPβ is an important early transcription factor that activates cell-cycle genes during mitotic clonal expansion (Reichert and Eick, 1999; Tang et al., 2003a). In addition, a recent study showed that the fat mass- and obesity-associated (FTO) gene affects adipogenesis by regulating mitotic clonal expansion (Merkestein et al., 2015). However, the underlying mechanism that regulates mitotic clonal expansion is poorly understood.

Sirtuins constitute a family of nicotinamide adenine dinucleotide (NAD)+-dependent protein deacetylases involved in stress resistance and metabolic homeostasis (Finkel et al., 2009). In mammals, there are seven members of this family (SIRT1–7) that localize to various subcellular compartments (Finkel et al., 2009). SIRT6 is found predominantly in the nucleus and plays a role in genomic stability, aging, glucose, and lipid metabolism, and the inflammatory response (Kawahara et al., 2009; Kim et al., 2010; Mostoslavsky et al., 2006; Xiao et al., 2010, 2012; Zhong et al., 2010). We have previously demonstrated that SIRT6 regulates glucose homeostasis and that SIRT6 deficiency results in severe hypoglycemia by enhancing insulin-stimulated glucose uptake in mice, leading to death of the majority of mice within 10 days after weaning (Xiao et al., 2010). Insulin increases glucose uptake and promotes the storage of substrates in fat, liver, and muscle by stimulating lipogenesis and glycogen and protein synthesis. However, SIRT6 mutant mice are very skinny and display a number of severe metabolic abnormalities (Xiao et al., 2010). The molecular mechanism regarding the role of SIRT6 in these processes is not fully understood.

We have shown that feeding SIRT6-deficient mice water containing 10% glucose could suppress their early post-weaning lethality, and most SIRT6-deficient mice (83%) survived to adulthood (Xiao et al., 2010), suggesting that energy homeostasis in SIRT6 mutant mice is impaired. Adipose tissue is an important metabolic organ that is crucial for whole-body insulin sensitivity and energy homeostasis. It has been reported that deletion of C/EBPα or C/EBPβ, which are essential for adipogenesis, impaired energy homeostasis and induced hypoglycemia in mice (Liu et al., 1999; Wang et al., 1995). SIRT6 deficiency also results in hypoglycemia and impairment of energy homeostasis, suggesting that SIRT6 could play an important role in regulating adipocyte differentiation. Using an adipogenesis assay, we demonstrate that SIRT6 deficiency impairs adipogenesis and that SIRT6 is an essential factor for mitotic clonal expansion during adipogenesis by inhibiting the expression of KIF5C and enhancing CK2 activity.

## RESULTS

### SIRT6 Is Required for Adipogenesis In Vivo and In Vitro

**Figure 1.**
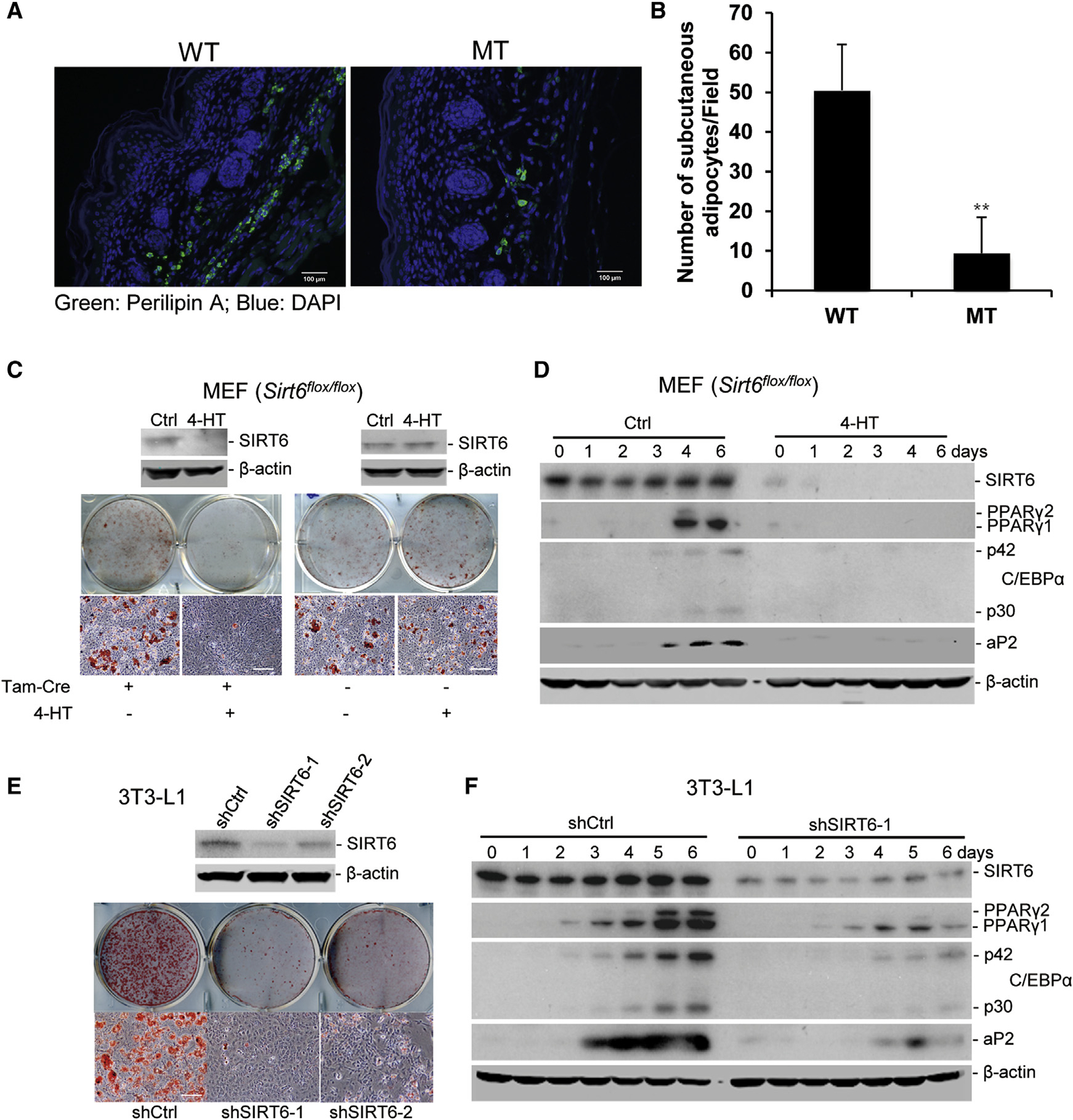
SIRT6 Deficiency Impairs Adipogenesis In Vivo and In Vitro (A) Representative immunofluorescence of subcutaneous adipocytes in WT and SIRT6 MT mice. n = 6 mice/genotype. Scale bars, 100 μm. (B) Quantification of (A). The number of perilipin A-positive cells in the subcutaneous area of six adjacent scapula sections was counted and averaged. One-way ANOVA was used for the statistical analysis. Data are represented as mean ± SD. **p < 0.01. (C and D) SIRT6 deficiency impairs adipogenesis of MEFs. *Sirt6*^*flox/flox*^ MEFs with or without Tam-Cre were treated with 4-HT for 2 days and induced with adipogenesis medium (day 0). (C) Morphological differentiation at 10 days. Top: western blot analysis of SIRT6 level. Center: stained plates. Bottom: representative fields under the microscope. Scale bars, 50 μm. (D) Western blot analysis of the expression of adipogenesis markers and SIRT6 during adipogenesis. (E and F) SIRT6 KD represses adipogenesis of 3T3-L1 cells. 3T3-L1 cells were infected with lentivirus-shSIRT6. Adipogenesis was induced after infection for 2 days (day 0). (E) Morphological differentiation at 8 days. Scale bars, 50 μm. (F) Western blot analysis of the expression of adipogenesis markers and SIRT6 during adipognesis.

To address the effect of SIRT6 on adipogenesis, we analyzed body weight and subcutaneous adipocytes in the SIRT6 mutant (MT) and wild-type (WT) neonatal pups (within 12 hr after birth). For analyzing the subcutaneous white adipocytes, transverse sections of the mice were cut at the level of the scapulae. Staining with an antibody against perilipin A, a protein that localizes on the membrane of lipid droplets of adipocytes (Brasaemle et al., 2004), we found that the number of subcutaneous adipocytes was remarkably reduced in the SIRT6 MT pups compared with their WT littermates (Figures 1A and 1B; Figure S1A), and the body weight of MT pups was lighter than that of WT pups (Figure S1B). These data suggest, but do not prove, that SIRT6 deficiency impaired fat development because SIRT6 is also mutated in other tissues that may indirectly affect adipogenesis. To further investigate the role of SIRT6 in adipogenesis, we used *Sirt6*^*flox/flox*^ mouse embryonic fibroblasts (MEFs) with or without tamoxifen-inducible Cre (*Tam-Cre*) for adipogenesis in vitro. We showed that *Sirt6* could be deleted in the *Sirt6*^*flox*/*flox*^; *Tam-Cre* MEFs but not in *Sirt6*^*flox/flox*^ MEFs without *Tam-Cre* (as a control) upon 4-hydroxytamoxifen (4-HT) treatment (Figure 1C). In adipogenesis assays, approximately 20%–30% of primary WT MEFs differentiated into adipocytes, as determined by oil red O staining. In contrast, deletion of *Sirt6* resulted in a severe adipogenesis defect and significantly reduced the expression of adipogenesis markers, including PPARγ, C/EBPα, aP2, and adiponectin (Figures 1C and 1D; Figure S1C). To confirm the function of SIRT6 in adipogenesis, we studied 3T3-L1 cells, and the data indicated that SIRT6 deficiency impaired the differentiation of 3T3-L1 cells into adipocytes (Figures 1E and 1F; Figure S1D). These data indicate that SIRT6 is required for adipogenesis in vivo and in vitro.

### SIRT6 Deficiency Impairs Mitotic Clonal Expansion during Adipogenesis

**Figure 2.**
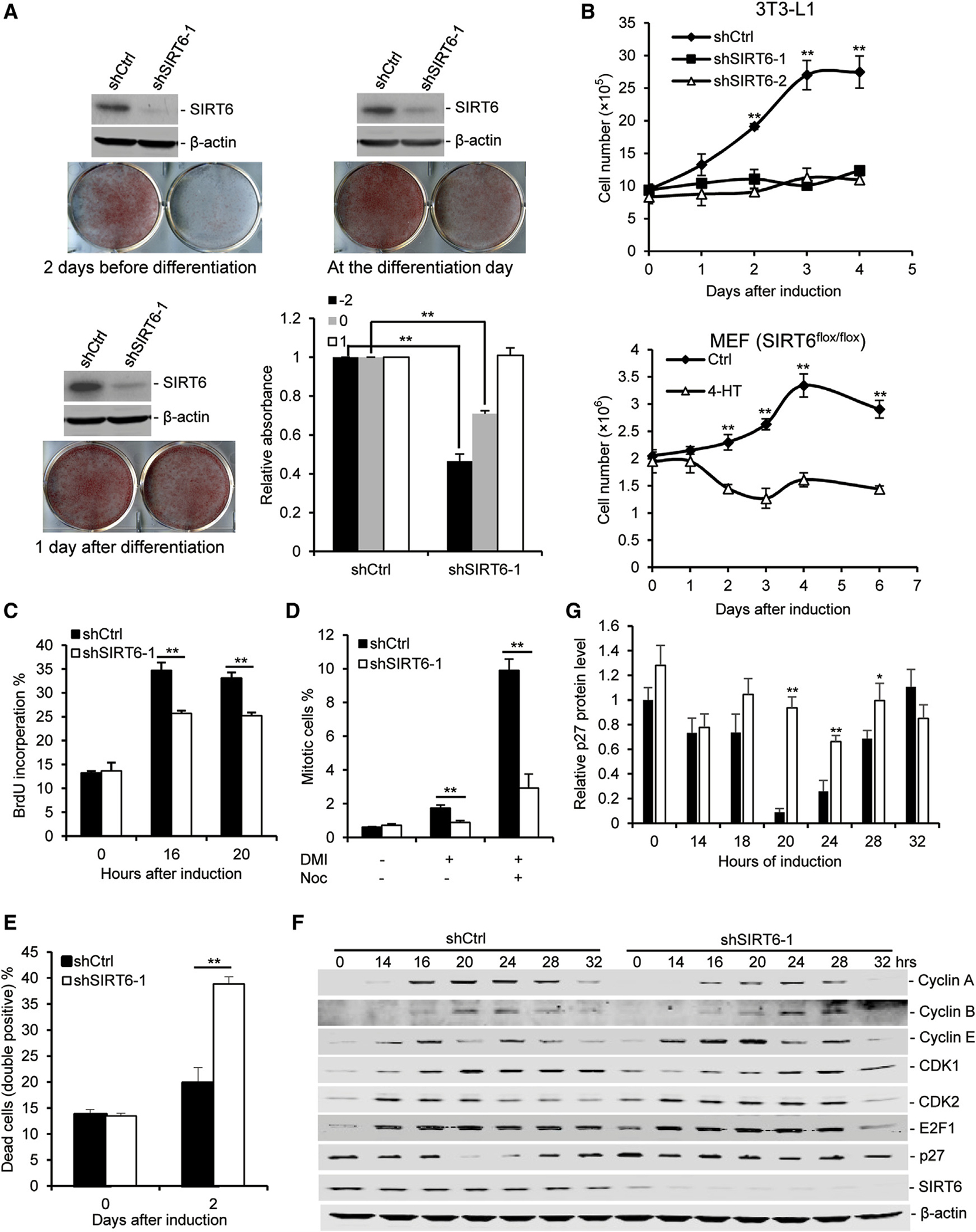
SIRT6 Deficiency Impairs Mitotic Clonal Expansion during Adipogenesis (A) SIRT6 KD represses adipogenesis at the early stage. Shown are representative western blots and stained plates of WT and SIRT6 KD cells. (B–G) SIRT6 deficiency suppresses cell proliferation during adipogenesis. (B) 3T3-L1 cells or MEFs were induced for adipogenesis. The cells were trypsinized from the plates at different time points and then counted by a cell viability counter (Cellometer Auto 2000). (C) BrdU incorporation of 3T3-L1 cells with or without DMI. 3T3-L1 cells were cultured for different times (0, 16, or 20 hr) and labeled by BrdU for 1 hr. Cells were fixed and subsequently stained for FACS analysis. (D) Percentage of mitotic cells with or without DMI. 3T3-L1 cells were cultured for 20 hr with or without DMI and continued to be culture for 4 hr in the presence or absence of Noc. Next, cells were fixed and subsequently stained for FACS analysis. (E) Percentage of cell death 2 days after induction. 3T3-L1 cells were cultured for 2 days with or without DMI, and cell death was measured by AnnexinV/PI staining. (F) Representative western blot of cell-cycle proteins and SIRT6 during adipogenesis in 3T3-L1 cells. (G) Quantifications of the p27 level from (F). The results were normalized by β-actin level. One-way ANOVA was used for the statistical analysis; n = 3/group. Data are represented as mean ± SD. **p < 0.01, *p < 0.05.

During adipogenesis, fibroblast-like preadipocytes differentiate into adipocytes through sequential, stages including growth arrest, mitotic clonal expansion, terminal differentiation, and maturation of white adipocytes (Lefterova and Lazar, 2009). To further investigate how SIRT6 regulates adipogenesis, 3T3-L1 cells were infected with short hairpin RNA (shRNA) targeting SIRT6 at different time points, followed by induction of adipogenesis. Knockdown (KD) of SIRT6 2 days before differentiation led to a severe adipogenesis defect (Figure 2A). KD of SIRT6 on the differentiation day partially blocked adipogenesis, whereas KD of SIRT6 1 day after differentiation had no effect on adipogenesis. The data suggest that SIRT6 is required for adipogenesis at the early stage.

One of the earliest events occurring in adipogenesis is that growth arrest preadipocytes undergo mitotic clonal expansion (Tang et al., 2003b). To determine whether SIRT6 deficiency affects mitotic clonal expansion, the number of 3T3-L1 cells and MEFs during the early process of adipogenesis was counted. The growth curve during adipogenesis showed that SIRT6 deficiency inhibited mitotic clonal expansion (Figure 2B). Preadipocytes cultured to confluence in vitro became growth-arrested at the G0-to-G1 cell cycle transition because of contact inhibition, and then they re-entered the cell cycle upon hormonal induction. A bromodeoxyuridine (BrdU) incorporation assay was performed in 3T3-L1 cells before and after induction for 16 and 20 hr to monitor this process. As shown in Figure 2C, the percentage of BrdU incorporation in cells treated with control shRNA increased BrdU-positive cells up to about 35% after induction for 16 and 20 hr, whereas SIRT6 KD cells had significantly less BrdU incorporation. Because the number of SIRT6 KD cells did not increase during this period of time despite about 25% of them being positive for BrdU, we measured the percentage of mitotic cells, and the data indicated that the mitotic index in control cells increased from 0.62% to 1.75%, whereas very little increase was detected in SIRT6 KD cells during this period of 20 hr in the differentiation medium (DMI) (Figure 2D). To exclude the possibility that the much lower mitotic index of SIRT6 KD is caused by a faster rate of cells exiting from mitosis, we treated cells with nocodazole (Noc), which accumulates in cells at mitosis, and the we detected that 10.63% cells in the control group entered mitosis, whereas only 2.57% of SIRT6 KD cells entered mitosis. Meanwhile, we found that SIRT6 KD cells have more apoptosis compared with WT cells after differentiation in 3T3-L1 cells (Figure 2E). These observations suggest that SIRT6 deficiency blocks the cell cycle at the S and M phases, which induces cell death. This may account for the failed cell expansion at the early phase of adipogenesis. To further investigate this, we measured the expression of proteins that regulate cell-cycle progression, such as cyclin A, cyclin B, cyclin E, CDK1, CDK2, E2F1, and p27, in 3T3-L1 cells after induction. p27, a CDK inhibitor, decreased in WT cells during adipogenesis, but its level was still high in SIRT6 KD cells (Figures 2F and 2G), suggesting that SIRT6 could regulate mitotic clonal expansion through inhibiting p27 degradation during adipogenesis. Therefore, these data indicate that SIRT6 plays a vital role in mitotic clonal expansion during adipogenesis.

### SIRT6 Represses KIF5C Expression during Adipogenesis

**Figure 3.**
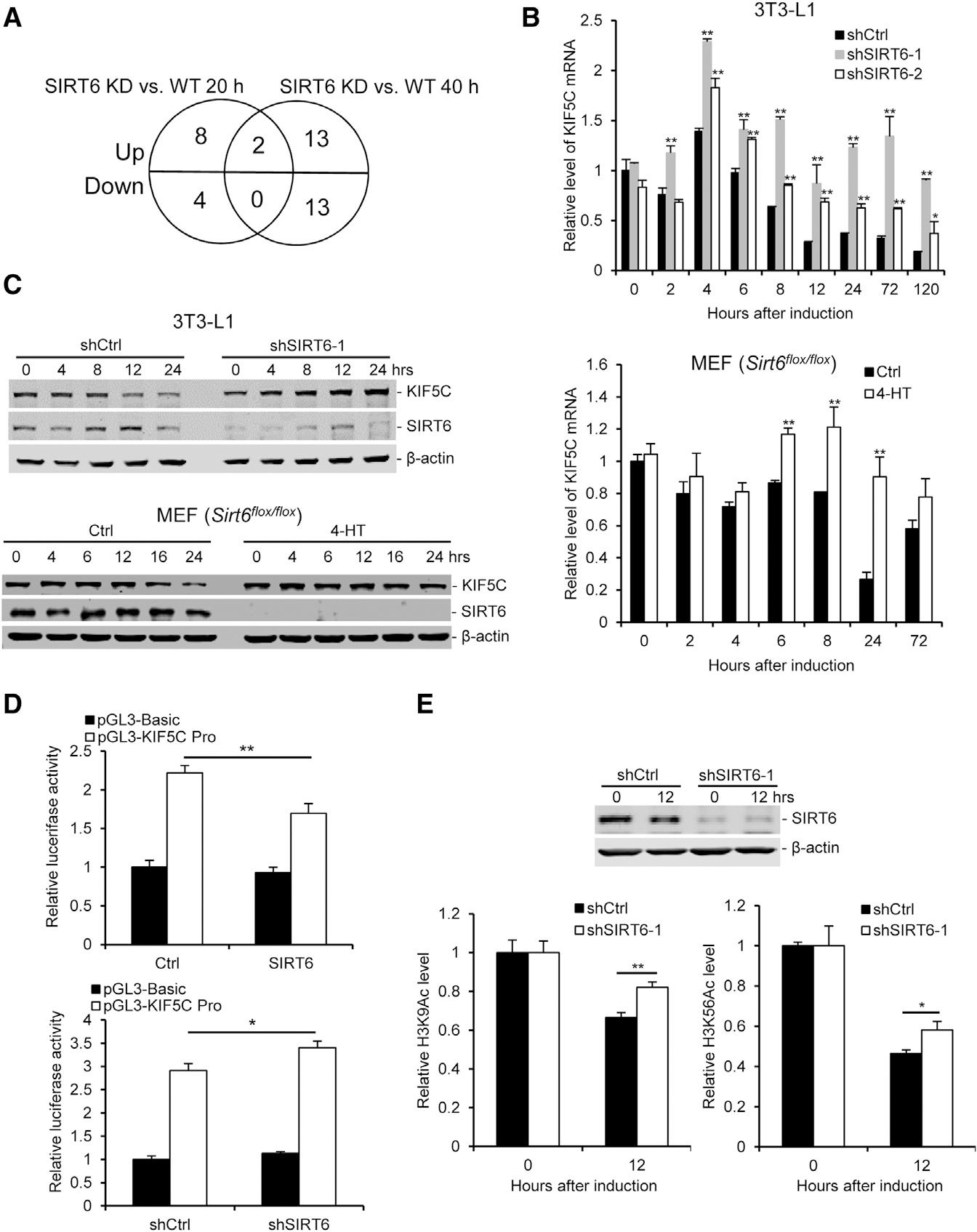
SIRT6 Negatively Regulates KIF5C Expression during Adipogenesis (A) Venn diagram showing that two common genes significantly changed when compared between the SIRT6 KD and WT group after induction for 20 or 40 hr. (B) The mRNA levels of the *Kif5c* gene were measured by real-time PCR during adipogenesis. Top: 3T3-L1 cells. Bottom: *Sirt6*^*flox/flox*^ MEFs. (C) Representative western blot of KIF5C and SIRT6 during adipogenesis. Top: 3T3-L1 cells. Bottom: *Sirt6*^*flox/flox*^ MEFs. (D) SIRT6 negatively regulates KIF5C promoter activity. A luciferase reporter driven by the KIF5C promoter (pGL3-KIF5C Pro) or a control reporter (pGL3-Basic) was transfected into 3T3-L1 cells with an SIRT6-expressing vector or shRNA against the SIRT6 vector, and luciferase activity was measured 24 hr after transfection. Top: OX of SIRT6. Bottom: KD of SIRT6. (E) SIRT6 regulates the levels of H3K9Ac and H3K56Ac at the promoter of KIF5C during adipogenesis. 3T3-L1 cells were cultured for 12 hr with or without DMI and then harvested for ChIP. Top: western blot analysis of the SIRT6 level after infection with shRNA for 2 days. Bottom: the levels of H3K9Ac and H3K56Ac. One-way ANOVA was used for the statistical analysis; n = 3/group. Data are represented as mean ± SD. **p < 0.01, *p < 0.05.

To further address how SIRT6 regulates mitotic clonal expansion during adipogenesis, we performed an mRNA microarray to measure gene expression in WT and SIRT6 KD 3T3-L1 cells before or after induction for 20 hr and 40 hr, respectively. After comparison of WT and SIRT6 KD cells, we found that ten genes were upregulated and four genes were downregulated in SIRT6 KD cells at 20 h, and and 15 genes were upregulated and 13 genes were downregulated in SIRT6 KD cells at 40 hr (Tables S1 and S2). Two genes, *Kif5c* and *Trim30d*, were commonly upregulated in SIRT6 KD cells at both time points (Figure 3A). Trim30d, also known as TRIM79α, is an interferon-stimulated gene product and is induced by virus infection (Taylor et al., 2011). Therefore, we focused on the function of KIF5C in adipogenesis. To validate the microarray data, the expression of KIF5C in 3T3-L1 cells and MEFs was measured at more time points after induction. The data revealed that KIF5C levels decreased during the course of adipogenesis in both WT cells but not in SIRT6 KD or acute knockout (KO) cells (Figures 3B and 3C), confirming that the expression of KIF5C during adipogenesis is inhibited by SIRT6.

KIF5C belongs to kinesin heavy-chain family that includes KIF5A and KIF5B (Kanai et al., 2000). To test whether SIRT6 especially regulates KIF5C expression during adipogenesis, we measured the expression of the other two members by real-time PCR and found that SIRT6 had no effect on their expression during adipogenesis (data not shown). Next, we constructed a *Kif5c* promoter (−634 to +240) luciferase reporter to address whether SIRT6 affects the activity of the *Kif5c* promoter. SIRT6 overexpression (OX) inhibited the luciferase activity driven by the *Kif5c* promoter but had no effect on the control reporter (Figure 3D). Likewise, SIRT6 KD increased the activity of the *Kif5c* promoter (Figure 3D). The results indicate that SIRT6 affects KIF5C expression through repressing the promoter activity of *Kif5c*.

It has been shown that SIRT6 regulates gene expression as a transcriptional co-repressor by deacetylating histone H3 lysine 9 (H3K9) and histone H3 lysine 56 (H3K56) at target gene promoters (Elhanati et al., 2013; Etchegaray et al., 2015; Kawahara et al., 2009; Kim et al., 2010; Michishita et al., 2009; Schwer et al., 2010). Therefore, the levels of acetylated H3K9 (H3K9Ac) and acetylated H3K56 (H3K56Ac) were measured in 3T3-L1 cells during adipogenesis by western blotting. The levels of H3K9Ac and H3K56Ac were higher in SIRT6 KD cells compared with WT cells after induction for 10 hr and 24 hr (Figures S2A and S2B), which suggests that SIRT6 regulates the levels of H3K9Ac and H3K56Ac during adipogenesis. To investigate whether SIRT6 affects H3K9Ac and H3K56Ac on the *Kif5c* promoter, we measured the recruitment of SIRT6 to the *Kif5c* promoter by chromatin immunoprecipitation (ChIP). The luciferase reporter assay showed that SIRT6 could affect the activity of the *Kif5c* promoter (Figure 3D), so we designed six primers for the *Kif5c* promoter according to the *Kif5c* promoter luciferase reporter (Figure S2C). The data showed that SIRT6 was recruited to the *Kif5c* promoter (−634 to −464) during adipogenesis (Figure S2D). We also found that the levels of H3K9Ac and H3K56Ac on the *Kif5c* promoter decreased as SIRT6 was recruited to the *Kif5c* promoter after induction (Figures S2E and S2F). Further result showed that the levels of H3K9Ac and H3K56Ac on the *Kif5c* promoter in WT cells were significantly lower than those in SIRT6 KD cells after induction (Figure 3E). Altogether, these data, at multiple levels, i.e., endogenous transcription of *Kif5c* (Figure 3B), luciferase reporter activity of the *Kif5c* promoter (Figure 3D), SIRT6 recruitment to the *Kif5c* promoter (Figure S2D), and reduced levels of H3K9Ac and H3K56Ac on the *Kif5c* promoter (Figure 3E) during adipogenesis, indicate that SIRT6 represses KIF5C expression during adipogenesis.

### KIF5C Negatively Regulates Adipogenesis

**Figure 4.**
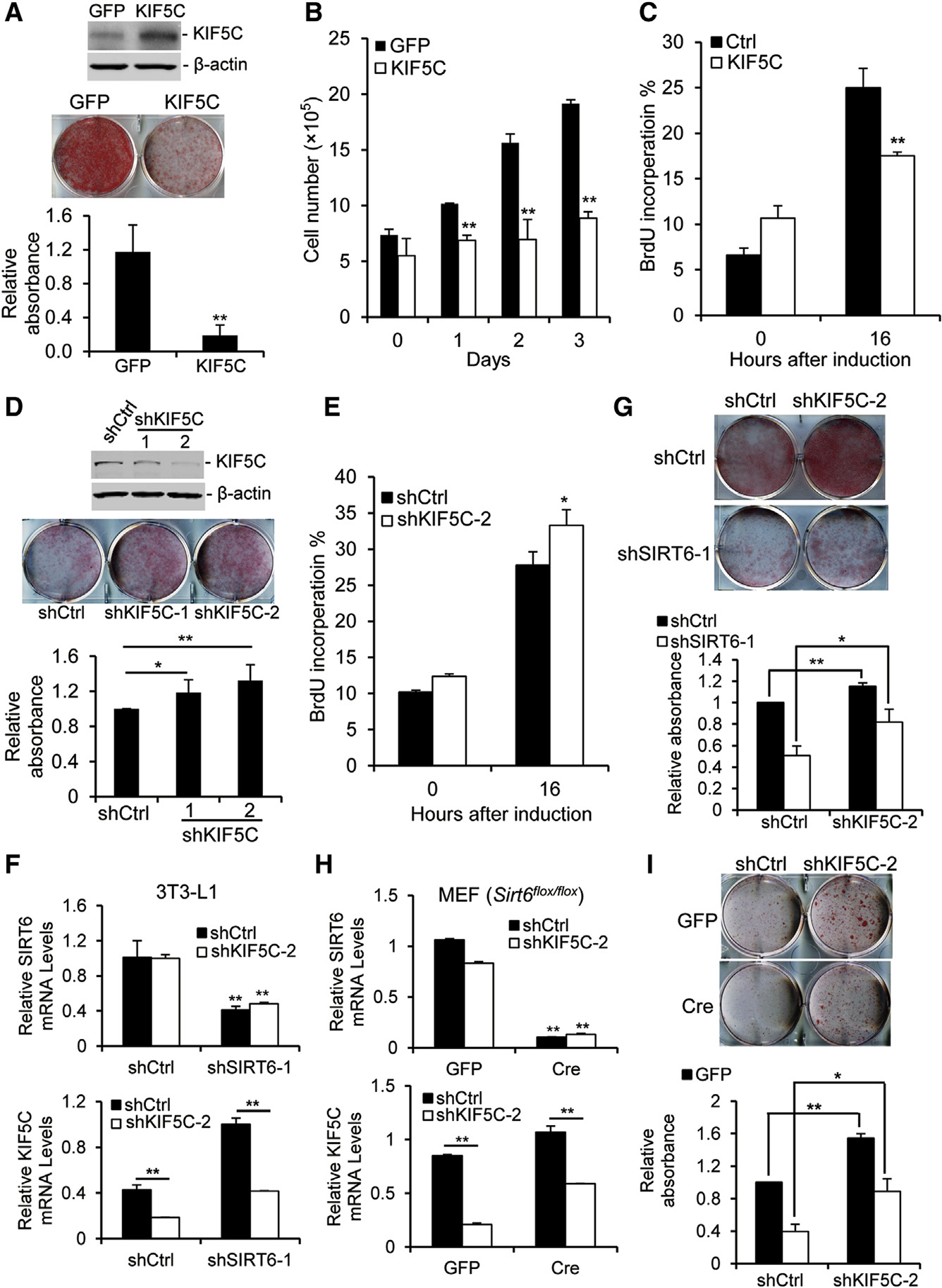
KIF5C Negatively Regulates Adipogenesis by Blocking Mitotic Clonal Expansion (A) OX of KIF5C represses adipogenesis. 3T3-L1 cells were infected with a retrovirus expressing KIF5C for 1 day and induced for adipogenesis. Top: western blot analysis of the KIF5C level. Center: stained plates. Bottom: the amount of oil red O was quantified. (B and C) OX of KIF5C inhibited cell proliferation during adipogenesis. 3T3-L1 cells were induced for adipogenesis after infection with a retrovirus expressing KIF5C for 1 day. (B) The cell number was counted during adipogenesis. (C) BrdU incorporation in the control and KIF5C OX groups before and after induction for 16 hr. (D) KIF5C KD enhances adipogenesis in 3T3-L1 cells. Top: western blot analysis of the KIF5C level. Center: stained plates. Bottom: the amount of oil red O was quantified. (E) KIF5C KD increases BrdU incorporation during adipogenesis. (F–I) KIF5C KD partially rescues adipogenesis in SIRT6-deficient cells. For 3T3-L1 cells, the cells were infected with lentivirus-shRNAs against SIRT6 and KIF5C for 2 days. For MEFs, the cells were infected with an adenovirus expressing Cre and lentivirus-shRNA targeting KIF5C for 2 days. (F and H) The SIRT6 and KIF5C mRNA levels in 3T3-L1 cells (F) or MEFs (H) were measured by real-time PCR. (G and I) Morphological differentiation and quantification of the oil red O amount at 8 days (G, 3T3-L1 cells) or 12 days (I, MEFs). One-way ANOVA was used for the statistical analysis; n = 3/group. Data are represented as mean ± SD. **p < 0.01, *p < 0.05.

KIF5C, as a neuronal form of KIF5, mainly expresses in lower motor neurons and maintains motor neurons (Kanai et al., 2000), but its function in adipogeneis is unknown. To investigate this, we performed an adipogenesis assay in KIF5C OX or KD 3T3-L1 cells. We found that KIF5C OX inhibited, whereas KIF5C KD enhanced, adipogenesis (Figures 4A and 4D), indicating that KIF5C negatively regulates adipogenesis. As mentioned above, SIRT6 could regulate mitotic clonal expansion and regulate the expression of KIF5C during adipogenesis. Therefore, we further investigated whether KIF5C regulates mitotic clonal expansion. The growth curve showed that KIF5C OX blocked cell proliferation during adipogenesis (Figure 4B). Furthermore, the BrdU incorporation assay showed that KIF5C OX significantly decreased BrdU incorporation in 3T3-L1 cells after induction for 16 hr compared with WT cells (Figure 4C), whereas KIF5C KD enhanced BrdU incorporation (Figure 4E). These results indicate that KIF5C, as a target of SIRT6, plays a vital role in mitotic clonal expansion and that SIRT6 deficiency impairs adipogenesis because of the increased KIF5C level in 3T3-L1 cells and MEFs.

To verify this, we investigated whether KIF5C KD could rescue the impaired adipogenesis in SIRT6 MT cells. 3T3-L1 cells were infected with shRNA against SIRT6 for 24 hr and then infected with shRNA-KIF5C. The results showed that the KIF5C level was higher in SIRT6 KD cells than in WT cells 24 hr after induction for adipogenesis, and KIF5C expression decreased to a level similar to that in WT cells after KD of KIF5C (Figure 4F). After knocking down KIF5C, adipogenesis in SIRT6 MT cells was partially rescued, and adipogenesis in WT cells was enhanced (Figure 4G). Similar results were obtained in MEFs carrying acute Cre-induced KO of *Sirt6*, where deletion of *Sirt6* increased the KIF5C level (Figure 4H), and KIF5C KD partially rescued adipogenesis (Figure 4I). These results demonstrate that SIRT6 negatively regulates adipogenesis, at least in part, mediated by KIF5C.

### KIF5C Interacts with CK2α′ and Inhibits CK2α′ Nuclear Translocation and Kinase Activity during Adipogenesis

**Figure 5.**
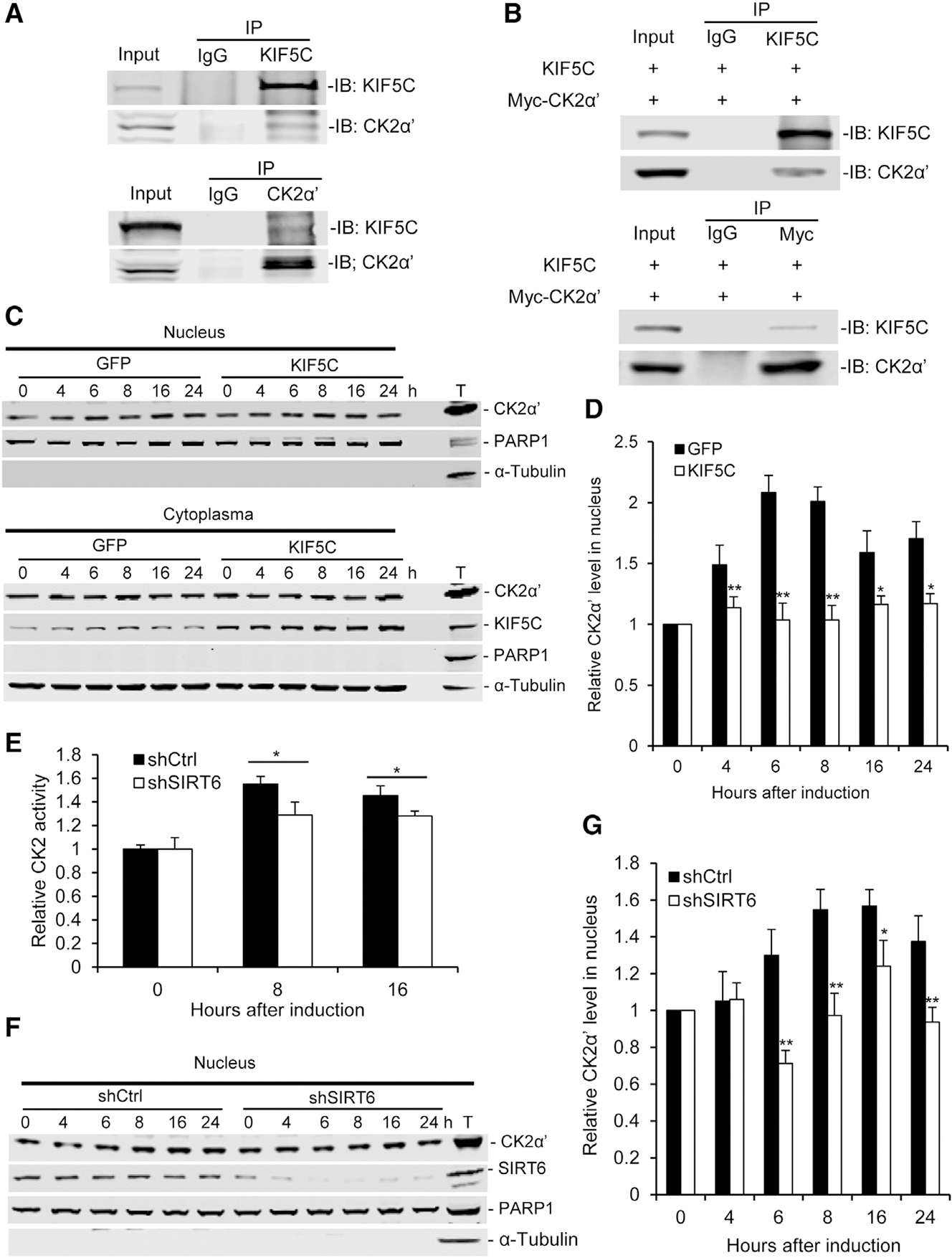
SIRT6 Deficiency Inhibits CK2 Activity during Adipogenesis by Blocking Nuclear Translocation of CK2α′ by KIF5C (A and B) KIF5C has interaction with CK2α′. (A) CoIP analysis of the interaction of endogenous KIF5C and CK2α′ in 3T3-L1 cells. (B) CoIP analysis of the interaction of KIF5C and CK2α′ in 293T cells transfected with KIF5C- and Myc-CK2α′-expressing vector. (C and D) The effect of KIF5C OX on nuclear translocation of CK2α′ during adipogenesis. (C) The CK2α′ level in the nucleus and the KIF5C level in the cytoplasm were analyzed by western blot. T, total lysate. (D) Quantification of the nuclear CK2α′ level in (C). The CK2α′level was normalized by the PARP1 level. (E) SIRT6 deficiency impairs CK2 activity during adipogenesis. The nuclear lysate was extracted from 3T3-L1 cells at different time points after adipogenesis. CK2 activity in nuclear lysate was measured by the kit and normalized by nuclear protein level. (F and G) SIRT6 KD blocks nuclear translocation of CK2α′ in 3T3-L1 cells. (F) The levels of CK2α′ and SIRT6 in the nucleus were measured by western blot analysis. T, total lysate. (G) Quantification of the nuclear CK2α′ level in (F). The CK2α′ level was normalized by the PARP1 level. One-way ANOVA was used for the statistical analysis; n = 3/group. Data are represented as mean ± SD. **p < 0.01, *p < 0.05.

Our results indicate that KIF5C negatively regulates adipogenesis through blocking mitotic clonal expansion. However, it is unclear how KIF5C, as a cytoplasm-based motor protein, executes this function. Recent studies showed that KIF5C interacts with CK2, which is a serine/threonine kinase with a multitude of substrates, including p27, and plays roles in cell differentiation, proliferation, and survival (Filhol and Cochet, 2009; Hauck et al., 2008; Schäfer et al., 2009). Our results showed that p27 degradation was blocked in SIRT6-deficient cells during adipogenesis. Thus, we hypothesized that KIF5C could affect cell proliferation during adipogenesis through regulating CK2 activity. To test this hypothesis, we investigated the interaction of KIF5C with CK2 in 3T3-L1 cells. CK2 mainly appears as a heterotetramer consisting of two catalytic α or α’ subunits and two regulatory β subunits (Filhol and Cochet, 2009). Co-immunoprecipitation (coIP) was performed to detect the interaction of endogenous KIF5C with CK2α and CK2α′. The data showed that KIF5C interacts with CK2α′. reciprocally but has no interaction with CK2α (Figure 5A; Figure S3A). This is perhaps due to the fact that KIF5C mainly localizes in the cytoplasm, where it serves as a motor protein for transporting specific cargoes along the microtubules (Schäfer et al., 2009), whereas CK2α mainly localizes in the nuclei of 3T3-L1 cells regardless of induction (Figure S3B). KIF5C and Myc-tagged CK2α′ were co-expressed in 293T cells, and we checked whether KIF5C has any interaction with CK2α′ under ectopic expression conditions. As shown in Figure 5B, KIF5C interacted with CK2α′ reciprocally, confirming this interaction in 3T3-L1 cells.

Furthermore, we investigated whether KIF5C has an effect on CK2 activity. The results showed that KIF5C KD enhanced CK2 kinase activity, whereas KIF5C OX impaired it (Figures S3C and S3D). Several lines of evidence have shown that a variety of growth stimuli evoke a translocation of CK2 to the nuclear matrix (NM), which is an important subnuclear locale of CK2 functional signaling (Guo et al., 2001; Tawfic et al., 2001; Yu et al., 2001). Our result showed that CK2 activity in nuclear lysate was significantly higher than in total lysate of 3T3-L1 cells (Figure S3E). Therefore, we hypothesized that KIF5C blocks CK2 activity by interacting with CK2α′ and inhibiting its nuclear translocation during adipogenesis. To investigate this, CK2α′ nuclear translocation was measured under KIF5C OX or KD conditions during adipogenesis by western blotting. CK2α′ went into the nucleus after induction for 6 hr, and KIF5C OX blocked CK2α′ nuclear translocation during adipogenesis (Figures 5C and 5D). In contrast, KIF5C KD significantly enhanced CK2α′ nuclear translocation 8 hr after induction (Figures S4A and S4B). These results indicate that KIF5C negatively regulates CK2 activity through blocking CK2α′ nuclear translocation. To further investigate whether SIRT6 could affect CK2 activity and CK2α′ nuclear translocation, we measured CK2 kinase activity in nuclear lysate and CK2α′ nuclear translocation before and after adipogenesis. The result showed that CK2 kinase activity increased in WT cells after induction for adipogenesis, whereas SIRT6 mutation impaired its activity (Figure 5E). The results also showed that SIRT6 KD impaired nuclear translocation of CK2α′ during adipogenesis (Figures 5F and 5G; Figures S4C and S4D). In addition, primary white preadipocytes were isolated from *Sirt6*^*flox/flox*^ mice and induced for adipogenesis. As shown in Figure S5, the results further confirm the conclusion that SIRT6 deficiency impairs adipogenesis and blocks nuclear translocation of CK2α′ by interaction with KIF5C.

### The Kinase CK2 Is Required for Mitotic Clonal Expansion during Adipogenesis

**Figure 6.**
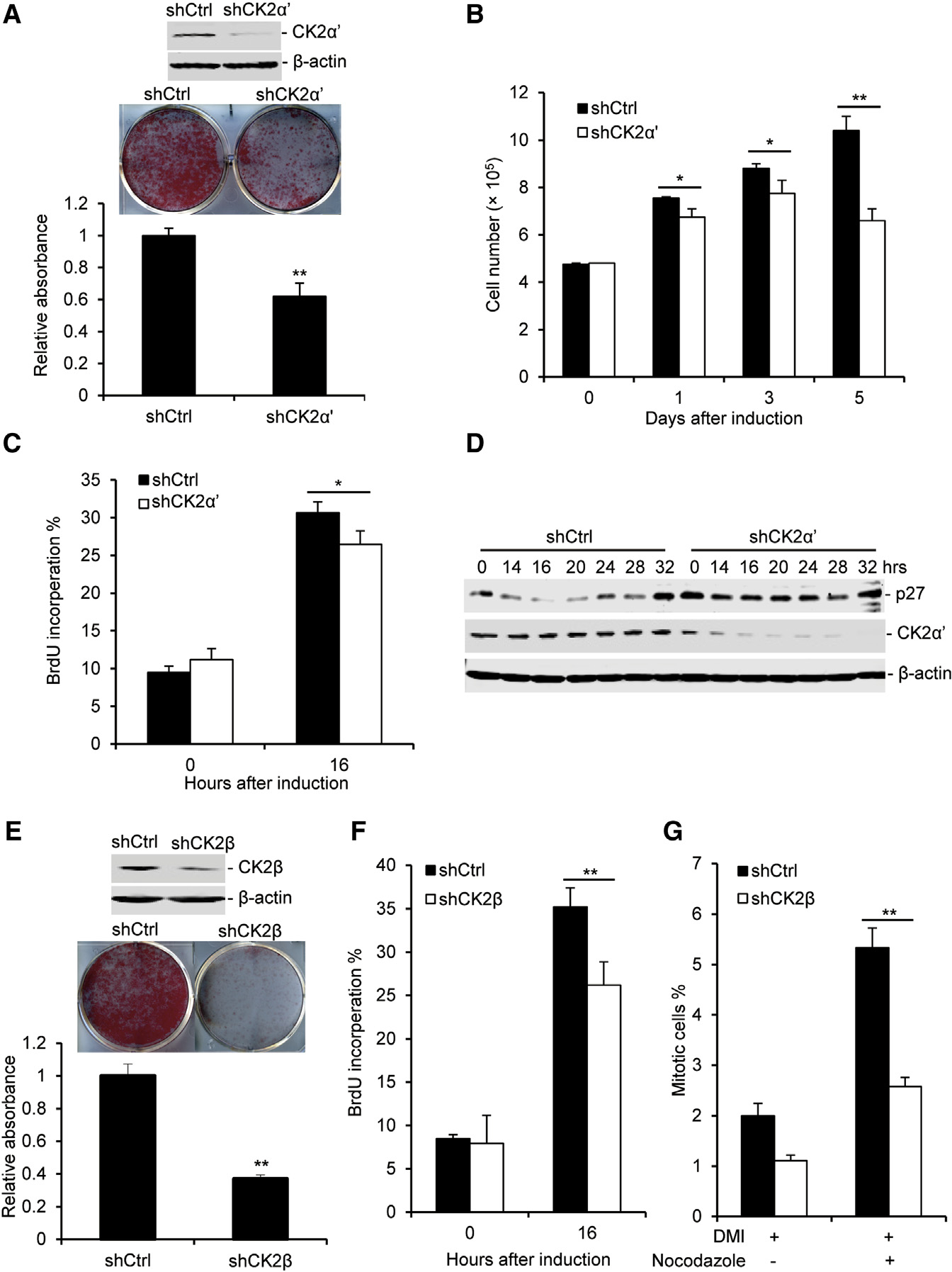
CK2 Kinase Is Essential for Adipogenesis (A) CK2α′ KD impairs adipogenesis in 3T3-L1 cells. Top: western blot analysis of CK2α′. Center: plates stained by oil red O. Bottom: the amount of oil red O was quantified. (B–D) CK2α′ KD inhibits cell proliferation during adipogenesis. (B) The cell number was counted at different time points after induction. (C) BrdU incorporation in the control and CK2α′ KD groups before and after induction for 16 hr. (D) Western blot analysis of p27 degradation during adipogenesis in 3T3-L1 cells (E) CK2β KD impairs adipogenesis in 3T3-L1 cells. Top: western blot analysis of CK2β. Center: plates stained by oil red O. Bottom: the amount of oil red O was quantified. (F and G) CK2β KD inhibits cell proliferation during adipogenesis. BrdU incorporation (F) and the percentage of mitotic cells (G) was measured by FACS analysis One-way ANOVA was used for the statistical analysis; n = 3/group. Data are represented as mean ± SD. **p < 0.01, *p < 0.05.

CK2 is involved in a variety of cellular processes, including cell-cycle progression, apoptosis, transcription, and viral infection (St-Denis and Litchfield, 2009). However, the function of CK2 in adipogenesis remains undetermined. Our results suggest that CK2 could play an important role in cell proliferation during adipogenesis. To investigate this, 3T3-L1 cells were infected with shRNA-CK2α′ for 2 days before they were subjected to an adipogenesis assay. KD of CK2α′ significantly impaired adipogenesis and blocked mitotic clonal expansion during adipogenesis (Figures 6A–6C). The data showed that SIRT6 KD inhibited CK2 kinase activity and p27 degradation during adipogenesis. It has been reported that, late in the G1 phase and early in S phase, p27 was phosphorylated at Thr187 by cyclin E-Cdk2 and that this phosphorylation promotes its proteasomal degradation (Carrano et al., 1999; Sheaff et al., 1997). A recent study showed that CK2 regulated the proliferation of cardiomyocytes by inducing p27 phosphorylation and degradation (Hauck et al., 2008). It has been suggested that SIRT6 could regulate CK2 activity and that CK2 mediates p27 degradation during adipogenesis. To test our hypothesis, the levels of p27 were detected in WT and CK2α′ KD cells during adipogenesis. As shown in Figure 6D, the p27 level significantly decreased in WT cells during adipogenesis but not in CK2α′ KD cells. Furthermore, the data showed that phosphorylated p27 (Thr187) was induced in WT 3T3-L1 cells during adipogenesis but not in CK2α′ KD or SIRT6 KD cells (Figure S6). Therefore, our data indicate that CK2 plays an important role in mitotic clonal expansion through inducing p27 phosphorylation and degradation and that SIRT6 promotes p27 degradation through the CK2 signaling pathway.

CK2β, as a regulatory subunit of CK2, affects the activity of catalytic subunits such as CK2α′ and CK2α (Niefind et al., 2001). To further confirm the role of CK2 in adipogenesis, KD of CK2β was performed in 3T3-L1 cells. As shown in Figures 6E–6G, CK2β KD resulted in a severe adipogenesis defect and inhibited mitotic clonal expansion. In addition, CK2 activity was blocked during adipogenesis by treating the cells with CK2 inhibitors such as quinalizarin and DMAT. Similarly, CK2 inhibitors also inhibited adipogenesis in 3T3-L1 cells and MEFs through blocking cell proliferation (Figure S7). These data indicate that CK2 is required for adipogenesis by inducing mitotic clonal expansion.

### Depletion of KIF5C Rescues the Phenotype in SIRT6 MT Mice

**Figure 7.**
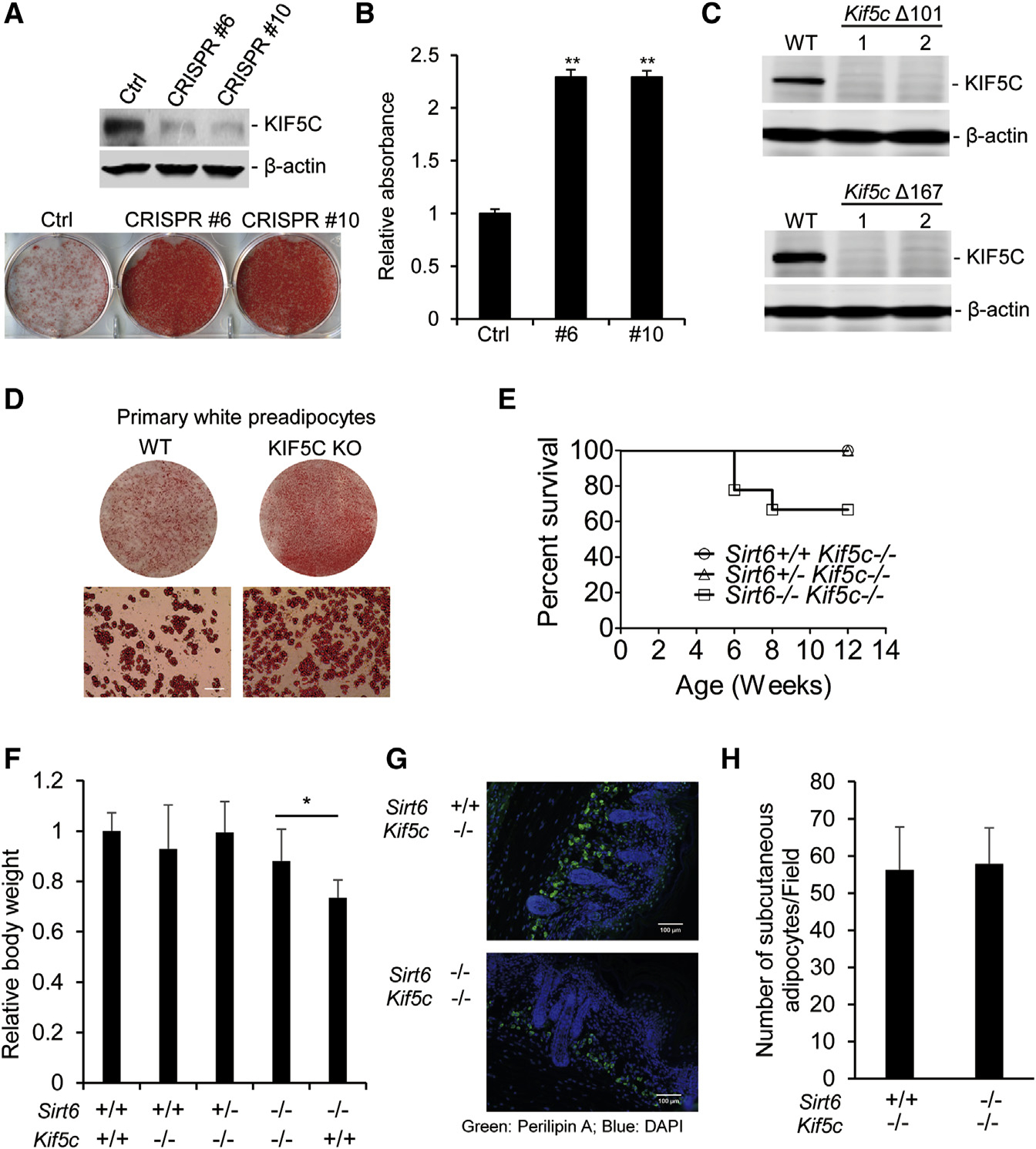
Deletion of Kif5c Partially Rescues Defects of SIRT6 MT Mice (A) KIF5C deficiency enhances adipogenesis of 3T3-L1. (B) The amount of oil red O in (A) was quantified. (C) Establishment of KIF5C MT mice. Shown is western blot analysis of the KIF5C level in brain tissues from two different deletion Kif5c mice. *KIF5C* MT mice have the same genetic background as SIRT6 MT mice (D) KIF5C deficiency enhances adipogenesis of primary white preadipocytes. Top: stained plates. Bottom: representative fields under the microscope. Scale bars, 50 μm. (E) Survival profile of *Sirt6*^+/+^ *Kif5c*^−/−^ (n = 10), *Sirt6*^+/−^ *Kif5c*^−/−^ (n = 30), and *Sirt6* ^−/−^ *Kif5c*^−/−^ (n = 19) female and male mice. (F) Relative body weight of mice after birth for 0.5 day. *Sirt6*^+/+^ *Kif5c*^+/+^, n= 12; *Sirt6*^+/+^ *Kif5c*^−/−^, n = 10; *Sirt6*^+/−^ *Kif5c*^−/−^, n = 26; *Sirt6*^−/−^ *Kif5c*^−/−^, n = 12; *Sirt6*^−/−^ *Kif5c*^+/+^, n = 9. (G) Representative immunofluorescence of subcutaneous adipocytes in *Sirt6*^+/+^ *Kif5c*^−/−^ and *Sirt6*^−/−^ *Kif5c*^−/−^ mice. *Sirt6*^+/+^ *Kif5c*^−/−^, n = 8; *Sirt6*^−/−^ *Kif5c*^−/−^, n = 8). (H) Quantification of (G). The number of perilipin A-positive cells in the subcutaneous area of adjacent scapula sections was counted and averaged. One-way ANOVA was used for the statistical analysis. Data are represented as mean ± SD. **p < 0.01, *p < 0.05.

The upregulation of KIF5C in SIRT6 MT cells prompted us to examine the genetic interaction between SIRT6 and KIF5C. We designed CRISPR vectors targeting different exons of *Kif5c* and found that targeting vectors against exon 6 and exon 10 worked well in 3T3-L1 cells. KIF5C mutant cells were established by using both vectors, and the results showed that deletion of *Kif5c* significantly enhanced adipogenesis (Figures 7A and 7B). To establish *Kif5c* and *Sirt6* double KO mice, CRISPR vectors targeting exon 10 were injected into zygotes isolated from *Sirt6* heterozygous mice. We established two different trains of KIF5C MT mice, carrying 101-bp and 167-bp deletions in exon 10, respectively (Figure 7C). Both strains of KIF5C MT mice did not display any obvious abnormalities up to a study period of 10 months, but KIF5C deficiency could enhance adipogenesis of primary white preadipocytes (Figure 7D), which is consistent with the in vitro assay (Figure 4D).

To investigate whether deletion of *Kif5c* could rescue defects of SIRT6 MT mice, we introduced a KIF5C mutation into SIRT6 MT mice. *Sirt6*^+/−^;*Kif5c*^−/−^ mice were normal and were interbred to generate *Sirt6*^−/−^;*Kif5c*^−/−^ mice. Nineteen *Sirt6*^−/−^;*Kif5c*^−/−^ mice were used for observing the survival profile. As shown in Figure 7E, seven *Sirt6*^−/−^;*Kif5c*^−/−^ mice died immediate after weaning, which yielded a survival rate of 63%. A previous study showed that the survival rate of *Sirt6*^−/−^ mice was 38% immediately after weaning (Xiao et al., 2010), suggesting that *Kif5c* deletion had partially rescued *Sirt6*^−/−^ mice. To investigate whether deletion of *Kif5c* rescues adipogenesis in *Sirt6*^−/−^ mice, we measured subcutaneous white adipocytes and the body weight of new pups. The results showed that the absence of KIF5C rescued the reduced body weight of SIRT6 MT new pups (Figure 7F). In addition, *Kif5c* KO enhanced subcutaneous adipocyte differentiation in SIRT6 MT mice; the number of subcutaneous adipocytes in double MT mice was similar to that in SIRT6 WT mice (Figures 7G and 7H). Although KIF5C and SIRT6 MT new pups had comparable subcutaneous adipocytes, the body weight of these mice was still less than that of WT mice as they grew (data not shown), which suggests that SIRT6 MT mice could have other defects. A recent study indicated that *Sirt6* KO embryonic stem cells are skewed toward neuroectoderm development and away from endoderm and mesoderm development (Etchegaray et al., 2015), which could cause other defects besides failure of adipogenesis. Collectively, these data indicate that SIRT6 is essential for adipogenesis and that SIRT6 enhances CK2 activity, which plays a vital role in mitotic clonal expansion during adipogenesis by disrupting the interaction of KIF5C with CK2α′.

## DISCUSSION

The sirtuins are an evolutionarily conserved family of NAD+-dependent deacetylases enzymes. SIRT6, as a member of sirtuin family, has a unique biological function in lipid metabolism, thus affecting diseases such as diabetes and obesity. Recent studies have shown that hepatic KO of *Sirt6* in mice results in fatty liver formation because of enhanced glycolysis and triglyceride synthesis (Kim et al., 2010), and hepatic-specific disruption of *Sirt6* also caused elevated serum cholesterol levels because of increasing the expression of the sterol-regulatory element binding protein (SREBP), a key regulator of cholesterol biosynthesis (Elhanati et al., 2013; Tao et al., 2013). However, this study showed that *Sirt6* mutant neonatal pups had fewer adipocytes, and *Sirt6*-deficient preadipocytes had a severe adipogenesis defect, indicating that SIRT6 plays an important role in regulating adipose development. Altogether, this study indicates that SIRT6 has distinct predominant functions in different tissues.

Adipogenesis occurs in several stages and involves a cascade of transcription factors (Lefterova and Lazar, 2009). The results showed that SIRT6 is involved in the mitotic clonal expansion stage but has no effect on the expression of transcription factors (C/EBPβ, C/EBPδ, KLF4, and Krox20) at the early stage (data not shown). We demonstrated that KIF5C, as a member of kinesin family, negatively regulates adipocyte differentiation, whereas the expression *KIF5C* is inhibited by SIRT6 through its deacetylation activity of H3K9 and H3K56. However, as shown in Figure 3D the effect of SIRT6 on KIF5C promoter activity is not very strong, suggesting that SIRT6 may also regulate KIF5C expression via mechanism(s) other than at the transcriptional level. In addition, although KIF5C deficiency promotes adipogenesis in primary white preadipocytes (Figure 7D), KIF5C KO mice did not show any obvious abnormalities. We think that the regulation of adipogenesis at the whole-organism level might be controlled by many factors and that the absence of KIF5C alone is not strong enough to affect it. On the other hand, KIF5C, as a brain-specific kinesin heavy chain, may have some unknown functions besides regulating adipogenesis. To further address this in the future, we will generate adipose-specific KIF5C KO mice.

Growth-arrested preadipocytes re-enter the cell cycle and undergo mitotic clonal expansion. The results showed that p27 was degraded in 3T3-L1 cells after induction for 20 hr but had no change in SIRT6 MT cells. The results also showed that CK2 activity was inhibited in SIRT6 MT cells during adipogenesis because of disruption of CK2α′ nuclear translocation by KIF5C. CK2, a serine/threonine kinase with a multitude of substrates, phosphorylates many proteins with important functions in the cell cycle (Meggio and Pinna, 2003). We found that CK2α′ KD and SIRT6 KD block p27 phosphorylation and degradation during adipogenesis, indicating that p27 degradation is regulated during adipogenesis by CK2, whose activity is impaired in SIRT6 MT cells.

Obesity is a highly prevalent condition in Western societies and a growing problem in developing countries. In this study, we demonstrated that SIRT6 plays a vital role in adipogenesis through regulating CK2 activity. Recent studies found several selective SIRT6 inhibitors that specifically block SIRT6 deacetyalation activity (Kokkonen et al., 2012; Parenti et al., 2014; Sociali et al., 2015). In addition, we found that CK2-selective inhibitors could block adipogenesis. Therefore, our study indicates a biological function of SIRT6 in adipogenesis and provides potential therapeutic targets for obesity.

## EXPERIMENTAL PROCEDURES

### Isolation of Primary Cells, Cell Culture, and Virus Infection

The 3T3-L1 preadipocyte cell line was cultured in DMEM plus 10% bovine serum (Sigma). 3T3-L1 cells were acquired from the ATCC. The ATCC authenticates the cell line before distribution and also tests for and confirms that all cell lines are free of mycoplasma contamination. Primary MEFs were isolated from E13.5 *Sirt6*^*flox/flox*^ embryos with or without Tam-Cre. Primary white preadipocytes were isolated from inguinal white adipose tissue (WAT) from 4- to 8-week-old *Sirt6*^*flox/flox*^ mice following a published protocol (Cho et al., 2009).

Lentivirus and retrovirus infection was done as described. Adenoviruses expressing Cre recombinase and GFP (Ad-Cre) or GFP alone (Ad-GFP) were purchased from the Vector Development Laboratory, Baylor College of Medicine. Adenovirus infection of primary MEFs and white preadipocytes that had a limited lifespan in culture were done at 100 MOI as described previously (Cho et al., 2009).

### Adipogenesis Assay

For adipogenesis of 3T3-L1 cells, differentiation was induced 2 days after the cells reached confluence by adding 0.5 mM 3-isobutyl-1-methylxanthine (IBMX), 1 μM dexamethasone (DEX), and 10 μg/ml insulin to medium containing 10% fetal bovine serum (FBS). After 2 additional days, the medium was replaced by DMEM and 10% FBS containing insulin, and then the media were changed every 2 days until the cells became mature adipocytes. For adipogenesis of primary MEFs, 2 days after cells reached confluence, cells were treated with medium supplemented with 0.5 mM IBMX, 1 μM DEX, 10 μg/ml insulin, and 0.5 μM rosiglitazone. Two days later, cells were changed to medium containing insulin and rosiglitazone. The medium was replenished at 2-day intervals for 12 days. Adipogenesis of primary white preadipocytes was carried out as described previously (Cho et al., 2009). To demonstrate adipogenesis, oil red O staining was used as described previously (Cho et al., 2009).

### Immunoprecipitation and Western Blot Analysis

3T3-L1 cells and 293T cells were lysed with lysis buffer 24 hr post-transfection. The cell lysates were immunoprecipitated by correspondent antibodies or control immunoglobulin G (IgG). After extensive washing, the precipitates were analyzed by western blot. The western blot was carried out by LI-COR Biosciences with correspondent antibodies. Quantifications of the western blot were performed using ImageJ (NIH).

### Immunofluorescence Staining

Methanol-fixed 3T3-L1 cells were stained with antibodies against CK2α′ or CK2α using methods described previously (Wang et al., 2004). Images were acquired using an LSM710 confocal microscope (Zeiss). Deparaffinized adjacent scapula sections from neonatal mice (within 12 hr after birth) were cooked with Retriever (catalog no. 62700–10, Electronic Microscopy Science) in buffer A (citrate buffer [pH 5.0]), followed by staining with antibody against perilipin A.

### Microarray and Real-Time PCR

Total RNA was isolated with Trizol reagent (Thermo Fisher Scientific) according to the manufacturer’s instructions. For the microarray analysis, RNA was processed using the RNeasy mini kit (QIAGEN) and analyzed using Mouse Gene 2.0 ST arrays (Affymetrix) at the Genomics Core in National Institute of Diabetes, Digestive and Kidney Diseases (NIDDK). Reverse transcription was performed using the QuantiTect reverse transcription kit (QIAGEN) Real-time PCR reactions were performed using SYBR Green PCR Master Mix (ABI). Relative quantification was achieved by normalization to the amount of 18S. The primers used for real-time PCR are listed in Table S3.

### ChIP

The ChIP assay was carried out as described previously (Wang et al., 2004). The ChIP primers for the KIF5C promoter are listed in Table S4.

### Transfection and Luciferase Assay

The transfections were carried out with Lipofectamine 2000 (Thermo Fisher Scientific) according to the manufacturer’s instructions. Cells were harvested 24 hr post-transfection, and luciferase activity was assayed using a dual luciferase reporter assay system (Promega).

### FACS Analysis

Fluorescence-activated cell sorting (FACS) analysis measuring BrdU incorporation or mitotic cells was carried out as described previously (Wang et al., 2014). For measuring BrdU incorporation, 3T3-L1 cells were cultured for the indicated times and labeled by BrdU for 1 hr. Cells were fixed and subsequently stained with propidium iodide (PI) and anti-BrdU conjugated with Alexa Fluor 488 for FACS analysis. For analyzing mitotic cells, 3T3-L1 cells were cultured for 20 hr with or without DMI and cultured for 4 hr in the presence or absence of Noc (200 ng/ml). Next, cells were fixed and subsequently stained with PI and anti-phospho-histone H3 (Ser10) conjugated with Alexa Fluor 488 for FACS analysis.

### CK2 Kinase Activity

CK2 kinase activity was measured by the CK2 assay kit (Millipore) according to the manufacturer’s instructions.

### In Vivo Study

All animal experiments were approved by the Animal Care and Use Committee (ACUC) of the NIDDK. SIRT6 mutant mice were generated as described previously (Xiao et al., 2010). KIF5C mutant mice were generated by CRISPR technology directly using embryos from SIRT6 mutant mice. The genetic background is 129SVEV/Black Swiss/FVB at roughly 1:1/2 ratio, which is the same as for the SIRT6 mutant mice we described earlier (Xiao et al., 2010). Neonatal pups (within 12 hr after birth), including male and female, were used for analyzing the subcutaneous white adipocytes.

For pronuclear injection (Ittner and Götz, 2007), we produced zygotes from *Sirt6* heterozygous mice. The CRISPR vector targeting KIF5C was purified by gel extraction and prepared by microinjection buffer. Next, the CRISPR vector (3 ng) was injected into a donor zygote, and injected zygotes were transfered into recipient foster mice. Finally, we screened the offspring and established *Kif5c* and *Sirt6* heterozygous mice.

### Statistical Analysis

All data shown represent the mean ± SD. At least three biological replicates were performed for all studies using cell cultures. All data were analyzed with one-way ANOVA, and p < 0.05 was considered statistically significant. All statistics were performed using SPSS 18.0.

## Supplementary Material

Supplemental information

## Highlights

- SIRT6 is essential for mitotic clonal expansion during adipogenesis
- SIRT6 negatively regulates KIF5C expression
- KIF5C is a negative regulator of mitotic clonal expansion
- KIF5C inhibits CK2 activity by blocking CK2α′ nuclear translocation during adipogenesis
