# Supplementary material for: SIRT6 Is Essential for Adipocyte Differentiation by Regulating Mitotic Clonal Expansion
Source: Cell Rep. Author manuscript; Available in PMC 2022 Aug 23. (PMC9396928; doi:10.1016/j.celrep.2017.03.006)
Supplement: Supplemental information [file NIHMS1828851-supplement-Supplemental_information.pdf]

Cell Reports, Volume *18*

Supplemental Information

SIRT6 Is Essential for Adipocyte Differentiation by Regulating Mitotic Clonal Expansion

Qiang Chen, Wenhui Hao, Cuiying Xiao, Ruihong Wang, Xiaoling Xu, Huiyan Lu, Weiping Chen, and Chu-Xia Deng

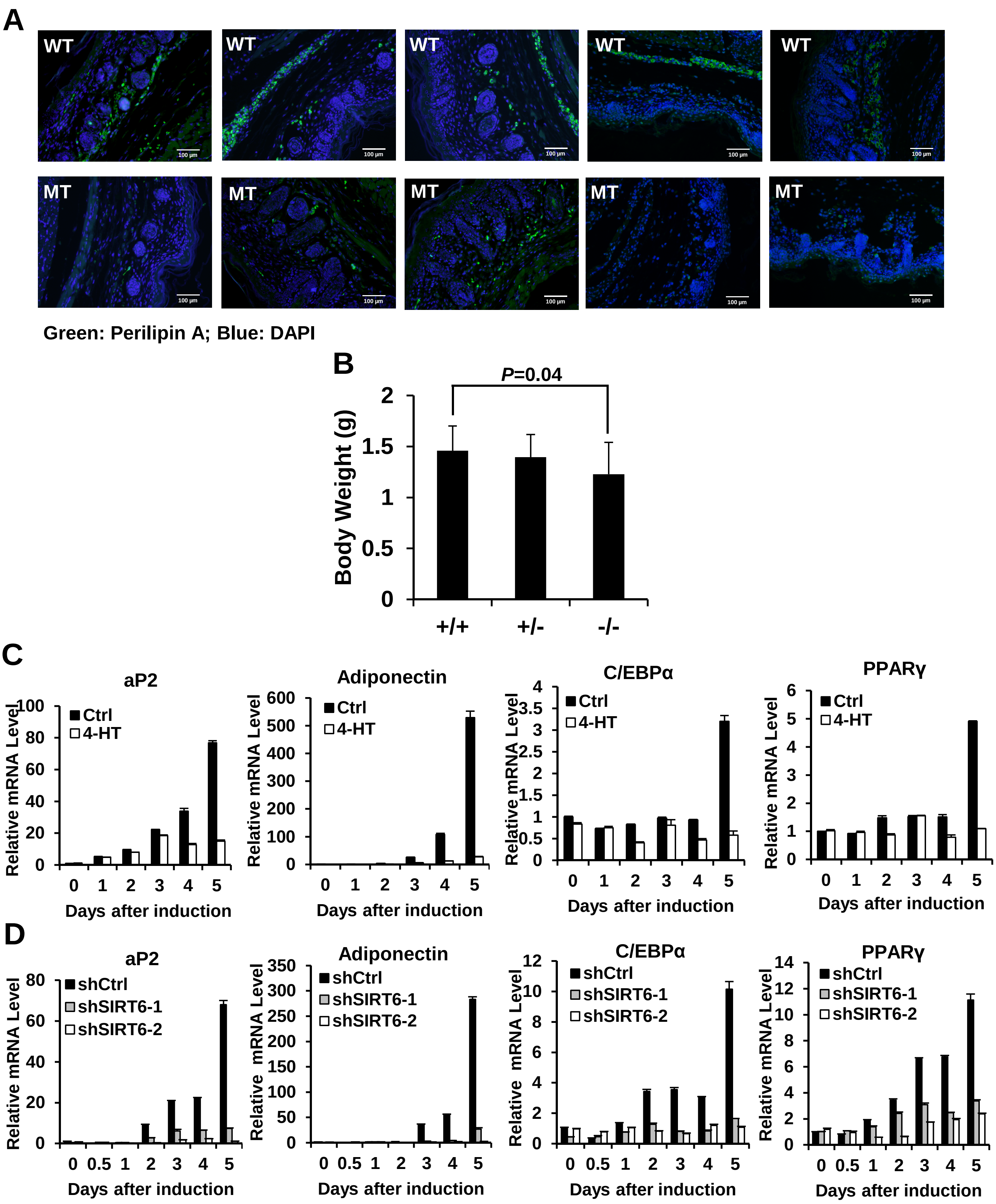

Figure S1. SIRT6 deficiency impairs adipogenesis *in vivo* and *in vitro* (related to Figure 1)

(A) Immunofluorescence of subcutaneous adipocytes in WT and MT mice. The scale bars represent 100 µm; (B) Body weight of mice after birth for 0.5 day. (Number of mice: $n_{+/+}$: 15; $n_{+/-}$: 25; $n_{-/-}$: 12); (C) The mRNA levels of adipogenesis markers in MEFs at different time points after induction; (D) The mRNA levels of adipogenesis markers in 3T3-L1 at different time points after induction.

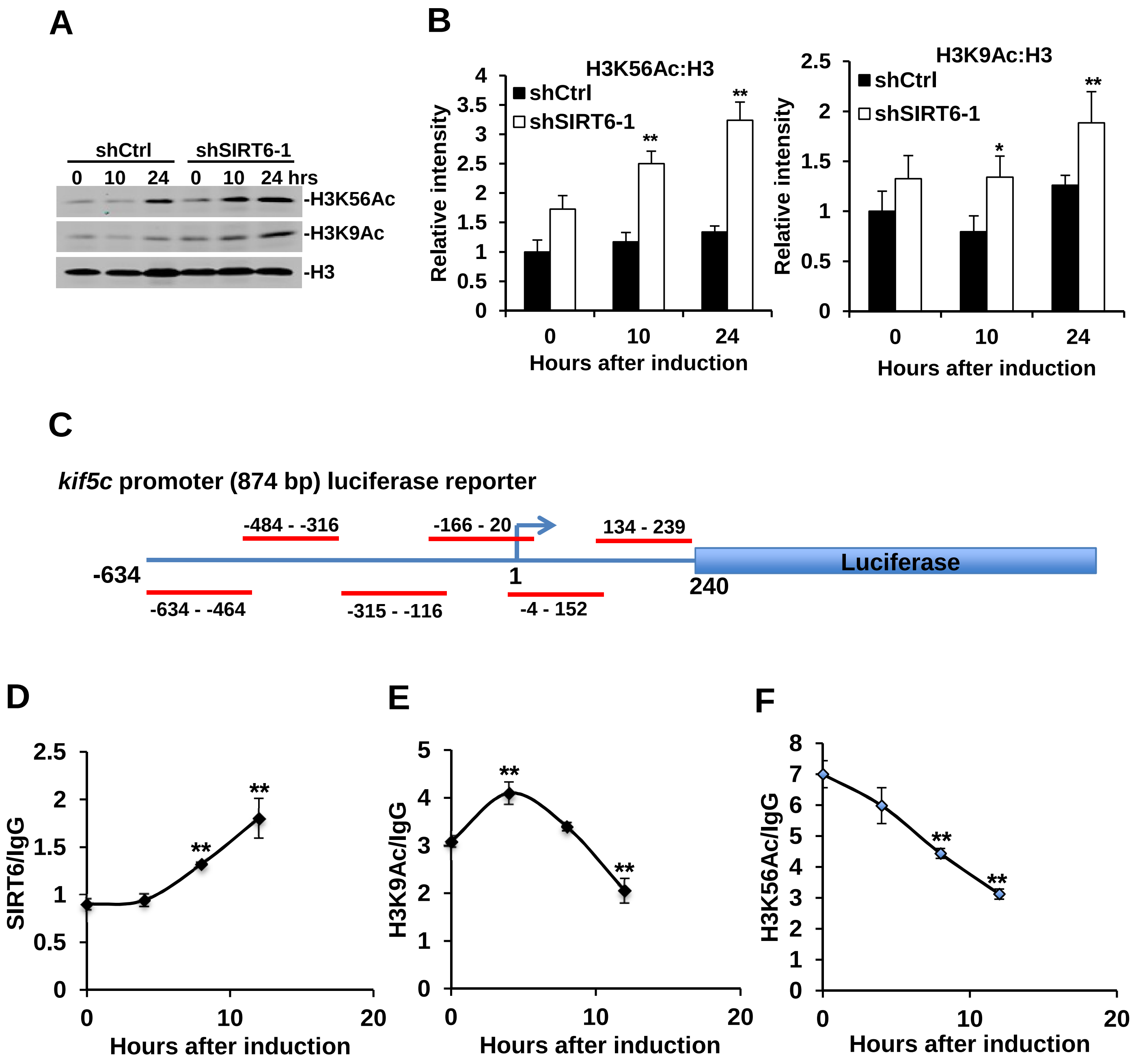

Figure S2. SIRT6 recruits on the promoter of KIF5C and mediates its activity through regulating acetylation of H3K9 and H3K56 during adipogenesis (related to Figure 3)

(A) Western blot analysis of H3K56Ac and H3K9Ac. Histone 3 (H3) is used as an internal control. 3T3-L1 cells were infected with Lentivirus-shRNA targeting SIRT6 for two days, and then induced for adipogenesis. Histone was extracted from the cells at different time points during adipogenesis; (B) Quantification of H3K56Ac and H3K9Ac level in (A); (C) The diagram of luciferase reporter driven by *Kif5c* promoter. The primers were designed for six fragments (labeled by red line) according to KIF5C luciferase reporter; (D-F) The levels of SIRT6 (D), H3K9Ac (E) and H3K56Ac (F) on the *Kif5c* promoter (-634 - -464) were measured by ChIP assay during adipogenesis (0, 4, 8, 12 h). One-way ANOVA test was used for the statistical analysis; n = 3 for each group. Data are represented as mean ± SD. ** P<0.01; * P<0.05.

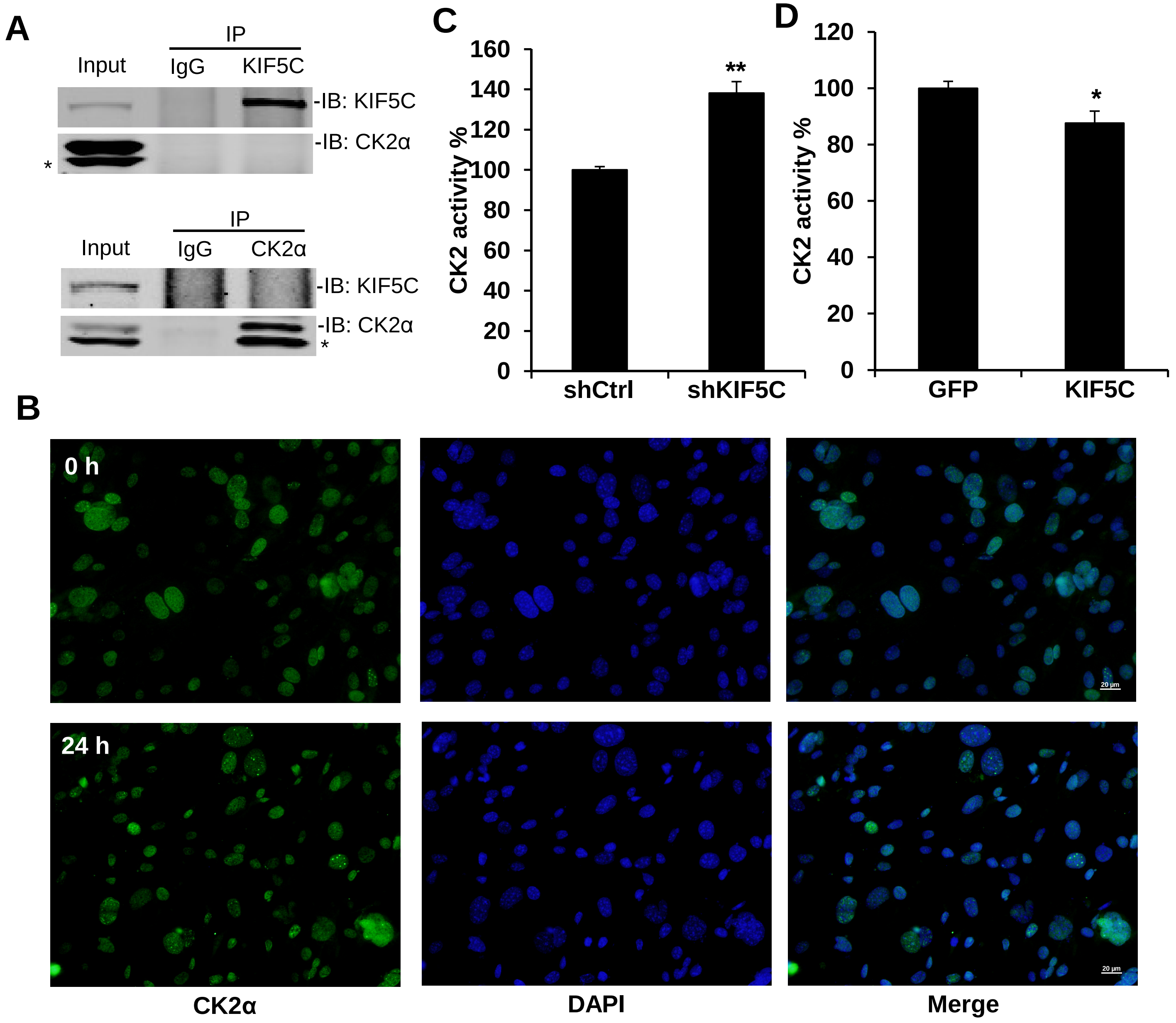

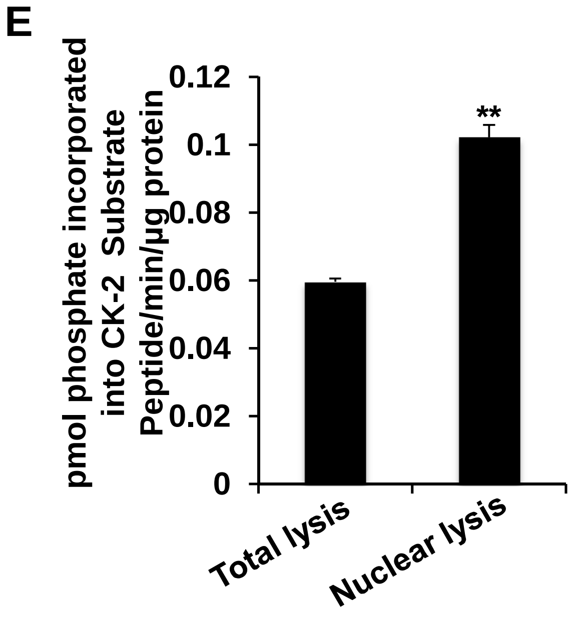

Figure S3. KIF5C inhibits CK2 kinase activity through interacting with CK2α', not with CK2α (related to Figure 5)

(A) Co-IP analysis of the interaction of endogenous KIF5C and CK2α in 3T3-L1 cells. * non-specific band; (B) Immunofluorescence of CK2α localization during adipogenesis. 3T3-L1 cells were fixed and stained by antibody against CK2α before or after induction for 24 h; The scale bars represent 20 μm; (C and D) KIF5C has effect on CK2 kinase activity. (C) KIF5C KD enhances CK2 kinase activity. CK2 kinase activity in 3T3-L1 cells was measured at two days after infected with Lentivirus-shRNA targeting KIF5C. (D) KIF5C OX impairs CK2 kinase activity. CK2 kinase activity in 3T3-L1 cells was measured at one day after infected with Retrovirus expressing KIF5C; (E) The CK2 kinase activity is higher in nuclear lysis than that in the total lysis. One-way ANOVA test was used for the statistical analysis; n = 3 for each group. Data are represented as mean ± SD. ** $P<0.01$; * $P<0.05$.

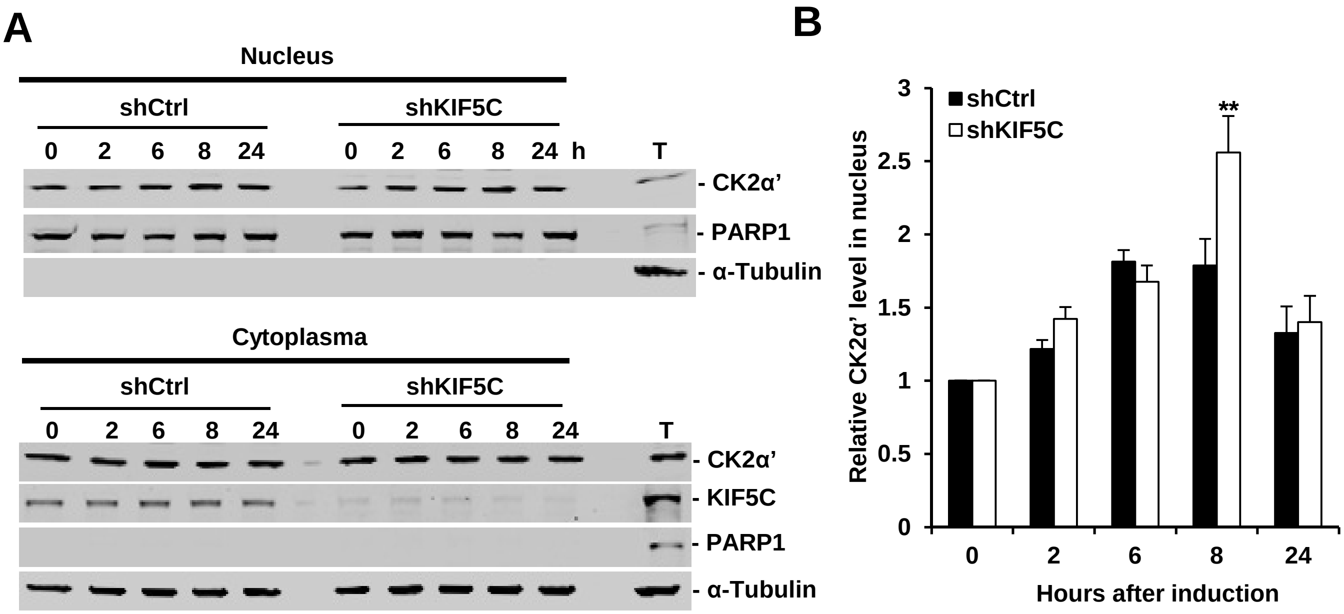

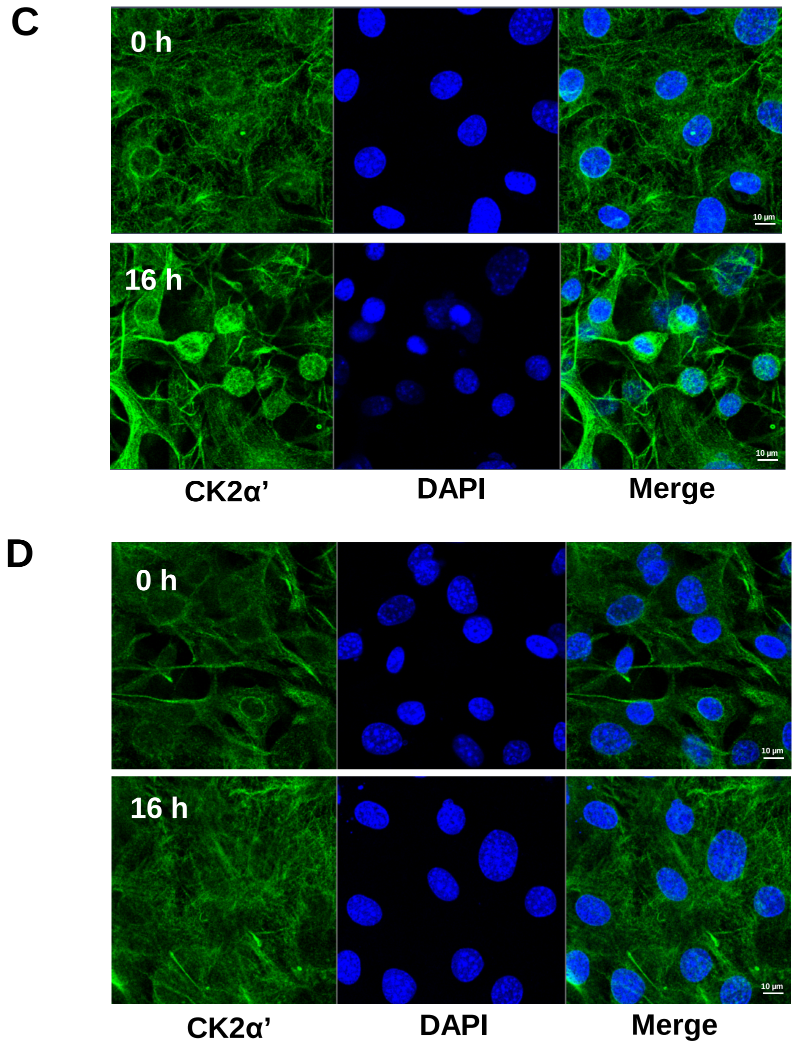

Figure S4. SIRT6 deficiency inhibits nuclear translocation of CK2α' during adipogenesis through regulating KIF5C expression (related to Figure 5)

(A and B) The effect of KIF5C KD on nuclear translocation of CK2α'. Nuclear protein and cytoplasmic protein were separated and extracted from the cells at different time points after adipogenesis. (A) The CK2α'level in nucleus and KIF5C level in cytoplasm were analyzed by Western blot. (B) Quantification of nuclear CK2α'level in (A). CK2α'level was normalized by PARP1 level. One-way ANOVA test was used for the statistical analysis; n = 3 for each group. Data are represented as mean ± SD. ** P<0.01;

(C and D) Immunofluorescence of CK2α' localization during adipogenesis. 3T3-L1 cells were fixed and stained by antibody against CK2α' before or after induction for 16 h. (C) 3T3-L1 infected with control shRNA. (D) 3T3-L1 infected with shRNA against SIRT6. The scale bars represent 10 μm.

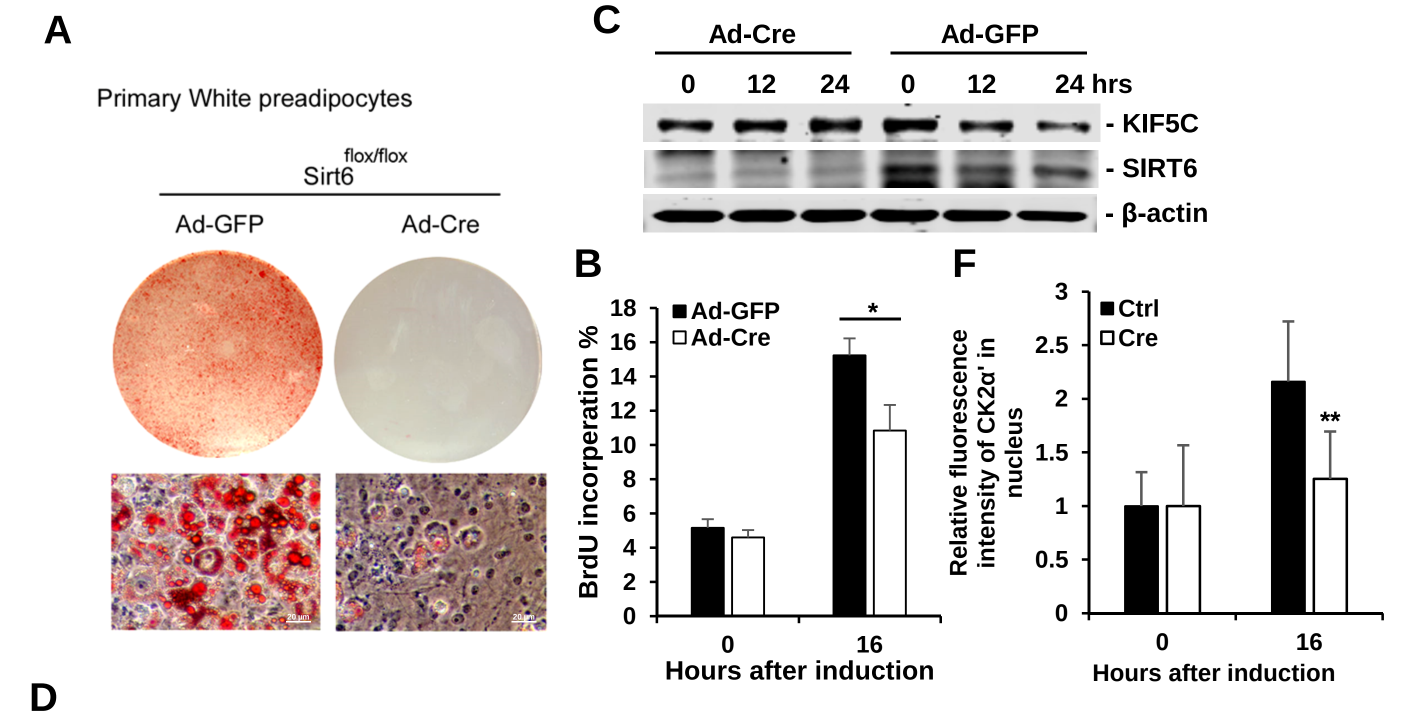

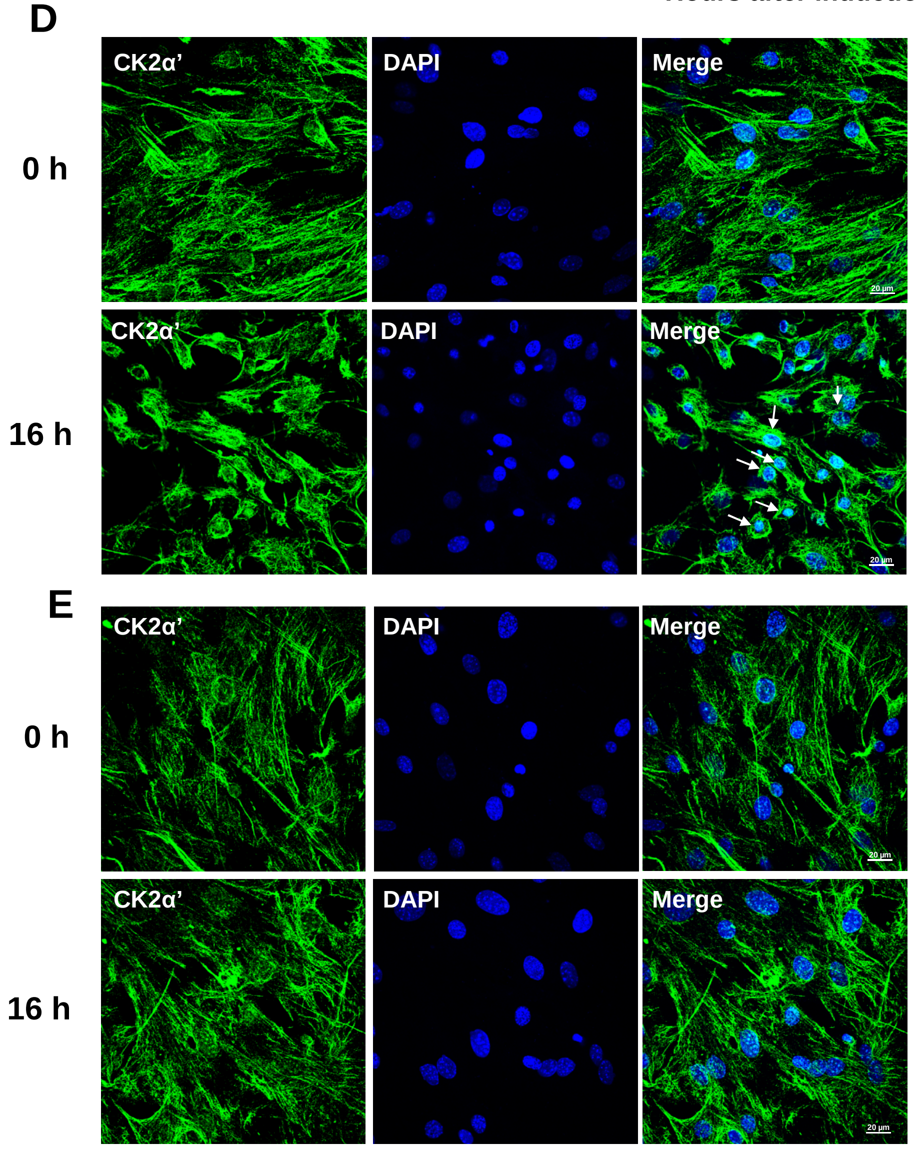

Figure S5. The effect of SIRT6 deficiency on adipogenesis of primary white preadipocytes (related to Figure1, Figure2 and Figure 5)

(A) SIRT6 deficiency impairs adipogenesis in primary white preadipocytes. The *Sirt6*$^{flox/flox}$ primary white preadipocytes were infected with Adenovirus-GFP (Ad-GFP) or Adenovirus-Cre (Ad-Cre) for two days. Next, the cells were induced for adipogenesis. Cells were stained by Oil Red O at 10 days after induction. Upper panels, stained plates; lower panels, representative fields under the microscope. The scale bars represent 20 µm; (B) BrdU incorporation in Ad-GFP and Ad-Cre group before and after induction for 16 h. * P<0.05.; (C) Western blotting of KIF5C and SIRT6 in primary white preadipocytes during adipogenesis; (D and E) Immunofluorescence of CK2α' localization during adipogenesis. Primary white preadipocytes were fixed and stained by antibody against CK2α' before or after induction for 16 h. (D) Control group, (E) Ad-Cre group. The arrow indicates CK2α' is translocated into nucleus. The scale bars represent 20 µm; (F) Quantification of nuclear CK2α' fluorescence intensity in (D) and (E) by Image J. At least 50 cells were measured in each group. ** P<0.01.

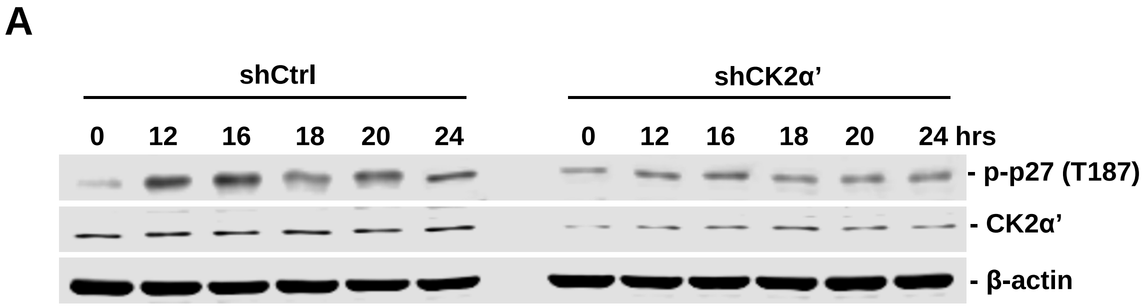

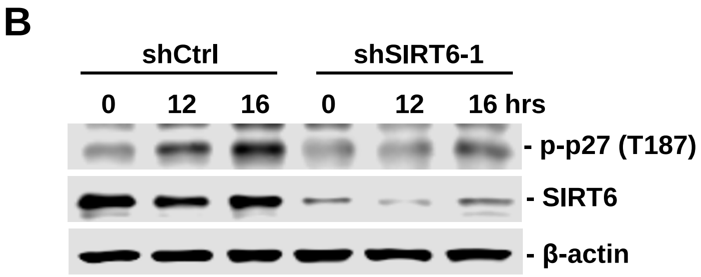

Figure S6. CK2α’ KD and SIRT6 KD impairs phosphorylation of p27 during adipogenesis (related to Figure 6)

(A) The effect of CK2α’ KD on phosphorylation of p27 (T187).

(B) The effect of SIRT6 KD on phosphorylation of p27 (T187).

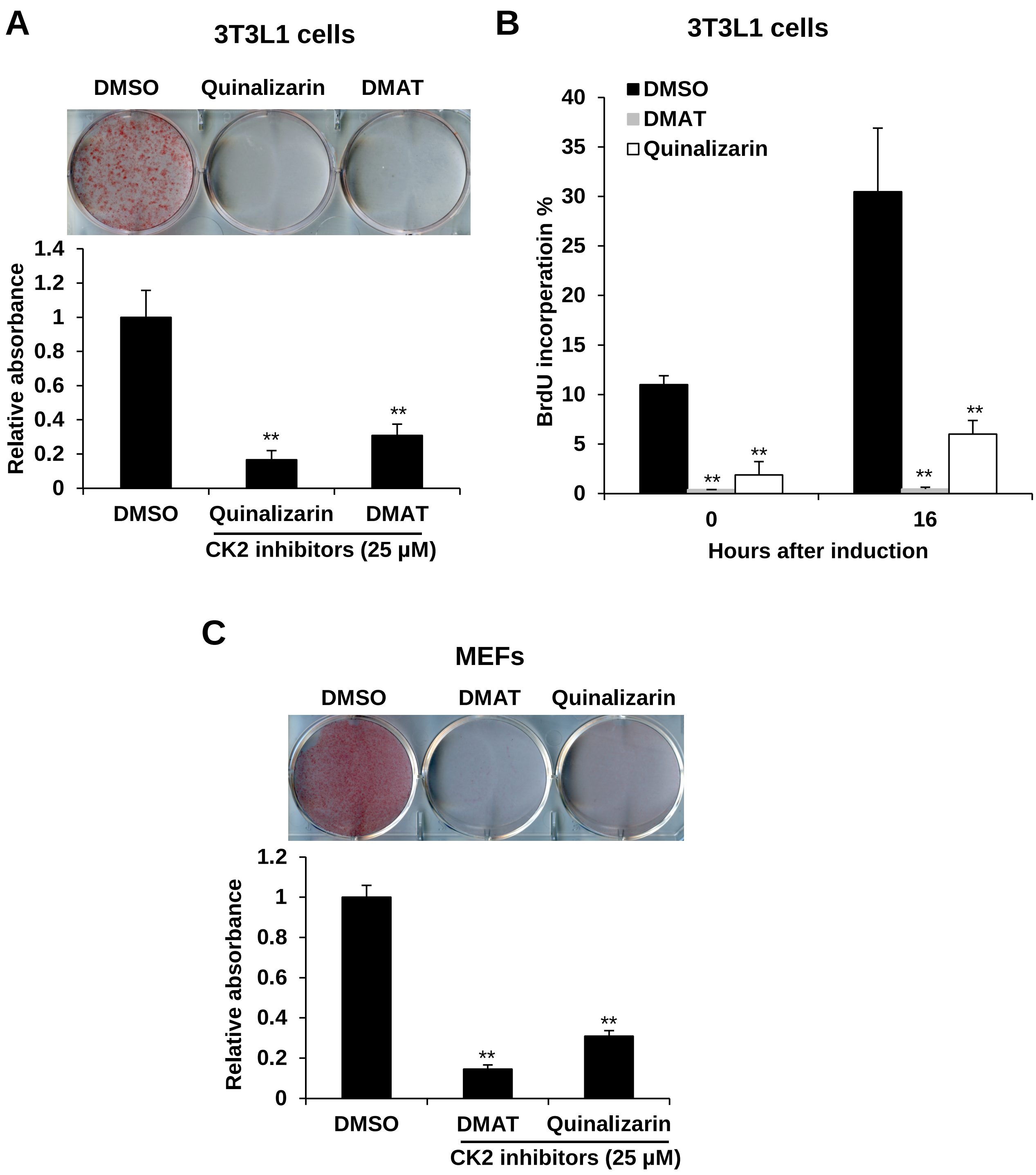

Figure S7. CK2 inhibitors blocks adipogenesis (related to Figure 6)

(A) 3T3-L1 cells were induced for adipogenesis with or without CK2 inhibitors (quinalizarin or DMAT). Upper panel, stained plates; lower panel, the amount of Oil Red O was quantified; (B) CK2 inhibitors blocks BrdU incorporation in 3T3-L1 cells during adipogenesis; (C) MEFs were induced for adipogenesis with or without CK2 inhibitors (quinalizarin or DMAT). Upper panel, stained plates; lower panel, the amount of Oil Red O was quantified. One-way ANOVA test was used for the statistical analysis; n = 3 for each group. Data are represented as mean $\pm$ SD. ** P<0.01.

Supplementary Table 1. Gene significantly changed in SIRT6 KD 3T3-L1 cells as compared to WT cells after induction for 20 h. Related to Figure 3

	**Gene Symbol**	**RefSeq**	**Fold-Change**	***p*-value**	**Function**
**Up**	*Npn2*	Z31359	1.768	0.01045	Cardiovascular development, axon guidance, and tumorigenesis
	Zfp71-rs1	NM_145622	1.60311	1.2E-05	Transcriptional regulation
	Vmn2r26	NM_019917	1.58876	0.03638	G-protein coupled receptor activity, response to pheromone
	Kif5c	NM_008449	1.56607	2.2E-05	Kinesin protein
	Nnat	NM_010923	1.56601	0.02641	Maintenance of the overall structure of the nervous system
	Defb7	NM_139220	1.55976	0.0053	Bactericidal activity.
	LOC100505096	XM_003688848	1.55492	0.01712	Unknown
	Trim30d	NM_199146	1.52153	0.00063	Involved in virus infection
	1110032F04Rik	NM_001167996	1.52019	0.01792	Integral component of membrane.
	Mir130b	NR_029659	1.50233	0.0165	Involved in post- transcriptional regulation of gene expression
**Down**	*Olfr1265*	NM_146343	-1.5536	0.00916	G-protein coupled receptor activity, olfactory receptor activity
	Enpp2	ENSMUST00000171545	-1.5712	1.1E-07	For generating lysophosphatidic acid (LPA)
	Olfr441	ENSMUST00000095954	-1.6443	0.01353	G-protein coupled receptor activity, olfactory receptor activity
	Vmn1r80	NM_134204	-1.7236	0.0073	Pheromone binding Source, pheromone receptor activity

Cut-off by fold-change >1.5 and *p*-value <0.05

Supplementary Table 2. Gene significantly changed in SIRT6 KD 3T3-L1 cells as compared to WT cells after induction for 40 h. Related to Figure 3

	Gene Symbol	RefSeq	Fold-Change	*p*-value	Gene function
Up	*Gm9125*	NM_001163730	2.55059	0.00346	Unknown
	Vmn1r180	NM_206869	1.87675	0.00561	Pheromone receptor activity
	Dpt	NM_019759	1.81294	5.48E-05	Involved in cell-matrix interactions and matrix assembly
	Hdx	ENSMUST00000113422	1.74669	0.01098	DNA binding
	Rab39b	NM_175122	1.65884	5.30E-06	Small GTPases that are involved in vesicular trafficking.
	Trim30d	NM_199146	1.56517	0.00037	Involved in virus infection
	Kif5c	NM_008449	1.5578	2.41E-05	Kinesin
	Gm5166	NR_027707	1.55325	0.0008	Unknown
	Ogn	NM_008760	1.55028	0.01114	Heparin binding, axonogenesis
	AF366264	NM_153093	1.54092	0.04627	Cysteine-type peptidase activity
	4930444P10Rik	ENSMUST00000115367	1.52064	0.01286	Spermatogenesis
	Vmn1r79	NM_001166835	1.51536	0.00109	Pheromone receptor activity
	D630023F18Rik	BC137870	1.50761	0.00201	Unknown
	Olfr810	ENSMUST00000091986	1.50728	0.00823	G-protein coupled receptor activity, olfactory receptor activity.
	Gm4841	NM_001034859	1.50575	0.00488	GTPase activity, central GTP binding
Down	*Entpd5*	NM_001026214	-1.50075	0.00023	Hydrolase activity, guanosine-diphosphatase activity
	Krt15	NM_008469	-1.50866	0.00336	Scaffold protein binding, structural molecule activity
	Brp44l	NM_018819	-1.52395	0.00605	Mitochondrial pyruvate transmembrane transport
	Nme2	ENSMUST00000072566	-1.5532	0.00122	Transcription factor
	Krt16	NM_008470	-1.55555	8.21E-05	Structural molecule activity and structural constituent of cytoskeleton
	Sft2d1	ENSMUST00000093169	-1.56511	0.02133	May be involved in fusion of retrograde transport vesicles derived from an endocytic compartment with the Golgi complex
	LOC100862386	XM_003688892	-1.61712	0.04266	Unknown
	Eif2s3x	NM_012010	-1.62341	0.00365	GTP binding and translation initiation factor activity
	Zfp862	NR_015597	-1.65415	4.38E-09	Nucleic acid binding and protein dimerization activity
	Nanp	NM_026086	-1.66102	0.02506	Involved in N-acetylneuraminate biosynthesis
	Efna2	NM_007909	-1.66202	3.43E-05	Ephrin receptor binding

Rps24	NM_011297	-1.66222	0.00633	Nucleotide binding, poly(A) RNA binding, structural constituent of ribosome
Rmrp	NR_001460	-1.66463	0.00015	Endoribonuclease, which cleaves mitochondrial RNA

Cut-off by fold-change >1.5 and *p*-value <0.05

Supplementary Table 3. Sequences used for real-time PCR. Related to Figure 3, Figure 4 and Figure S1

Gene	Forward primer (5’ - 3’)	Reverse primer (5’ - 3’)
Sirt6	AGTCCTCCAGCGTGGTTTT	AGCATTCTCGAAGGTGGTGT
Pparg	GCTGTTATGGGTGAAACTCT	TGGCATCTCTGTGTCAACCA
Cebpa	CAAGAACAGCAACGAGTACCG	GTCACTGGTCAACTCCAGCAC
Fabp4	ACACCGAGATTTCCTTCAAACTG	CCATCTAGGGTTATGATGCTCTTCA
Adipoq	GTGATGGCAGAGATGGCACT	CCTTCAGCTCCTGTCATTCC
Cebpb	AAGCTGAGCGACGAGTACAAGA	GTCAGCTCCAGCACCTTGTG
Cebpd	CGACTTCAGCGCCTACATTGA	CTAGCGACAGACCCCACAC
Kif5c	GCCTTGAAGAGTGTCCTCCA	GGGCTCTAGGCTCTTCTGGT
Kif5a	CATCCATTGTGGTACGCATC	CTCTTCCTGGTCCAGCATCT
Kif5b	CCAACAAAGCCAATTGGTAGA	AGGCGATTCAGTTCAGCTTG
Rn18s	AGTCCCTGCCCTTTGTACACA	CGATCCGAGGGCCTCACTA

Supplementary Table 4. Sequences used for ChIP assay. Related to Figure 3 and Figure S2

Name	Forward primer (5’ - 3’)	Reserve primer (5’ - 3’)
KIF5C ChIP-1	GCTGGAGGTCAGCAGCAG	CAGCTTCTCAGCAGGTCATC
KIF5C ChIP-2	GAGCACGGTGGTGCTGAT	CCTGCTGCTGACCTCCAG
KIF5C ChIP-3	GGGGGTCCTCTCAAGCTAAT	CATCCTGCATCAGCACCAC
KIF5C ChIP-4	ACAACACAGGGAAACGCTCT	GCAAGGCTTGGATTAGCTTG
KIF5C ChIP-5	TCTGACTTAGCGTTGCAGTGA	AGAGCGTTTCCCTGTGTTGT
KIF5C ChIP-6	TTTTGTCCCAAGTTGCCTTC	TCACTGCAACGCTAAGTCAGA

Experimental Procedures

Reagents, Plasmids and Antibodies.

All reagents used for cell culture were purchased from Invitrogen. CK2 inhibitors such as Quinalizarin and DMAT were purchased from Sigma. The lentiviral shRNA plasmid pLKO.1 targeting SIRT6 (clone ID TRCN0000108930, TRCN0000108934), KIF5C (TRCN0000311436, TRCN0000354039), CK2α' (TRCN0000025664), CK2β (TRCN0000278122), and shRNA control plasmid were purchased from Sigma. Mouse full-length KIF5C and CK2α' expressing vector were purchased from Origene (Rockville, MD), and KIF5C cDNA was cloned into retroviral plasmid pBabe-puro. pX330 (hSpCas9 + chimeric guide RNA) plasmid was requested from Feng Zhang's lab. The guide sequence targeting KIF5C was synthesized and inserted into pX330 by BbsI according to protocol from Zhang's lab.

Anti-Perilipin A (P1998), anti-β-actin (A5316), anti-α-Tubulin (T5168) and anti-SIRT6 (S4322) for ChIP were purchased from Sigma. Anti-PPARγ (2443), anti-C/EBPα (2295), anti-aP-2 (3544), anti-PARP1 (9532), anti-Akt (9272), anti-phospho-Akt (Ser473, 9271), anti-phospho-histone H3 (Ser10) (9706) and anti-SIRT6 (12486) for Western blot were from Cell signaling (Danvers, MA). Anti-CyclinA (sc-751), anti-CyclinB (sc-245), anti-CyclinE (sc-481), anti-CDK1 (sc-54), anti-CDK2 (sc-163), anti-E2F1 (sc-193), anti-p27 (sc-527), anti-p-p27 (Thr 187, sc-16324), anti-KIF5C (sc-134602), anti-CK2α' (sc-6481), anti-CK2α (sc-373894) and anti-c-Myc (sc-40) were from Santa Cruz Biotechnology, Inc. Anti-CK2β (04-1128), anti-Histone3 (06-755) were from Millipore (Billerica, MA). Anti-BrdU (347580) was from BD Biosciences. Anti-H3K9Ac (ab4441), anti-CK2α' (ab10474) was from Abcam (Cambridge, MA). Anti-H3K56Ac (A-4026-050) was from Epigentek Group Inc. (Brooklyn, NY).
